# Improving Phenotyping of Patients With Immune-Mediated Inflammatory Diseases Through Automated Processing of Discharge Summaries: Multicenter Cohort Study

**DOI:** 10.2196/68704

**Published:** 2025-04-09

**Authors:** Adam Remaki, Jacques Ung, Pierre Pages, Perceval Wajsburt, Elise Liu, Guillaume Faure, Thomas Petit-Jean, Xavier Tannier, Christel Gérardin

**Affiliations:** 1 Limics Université Sorbonne Paris-Nord, Inserm Sorbonne Université Paris France; 2 Pôle Innovation et Données Direction des Services Numériques Assistance Publique – Hôpitaux de Paris Paris France; 3 Centre de Pharmacoépidémiologie Hôpital Pitié Salpêtrière Assistance Publique – Hôpitaux de Paris Paris France; 4 Service de médecine interne Hôpital Tenon Assistance Publique – Hôpitaux de Paris Paris France

**Keywords:** secondary use of clinical data for research and surveillance, clinical informatics, clinical data warehouse, electronic health record, data science, artificial intelligence, AI, natural language processing, ontologies, classifications, coding, tools, programs and algorithms, immune-mediated inflammatory diseases

## Abstract

**Background:**

Valuable insights gathered by clinicians during their inquiries and documented in textual reports are often unavailable in the structured data recorded in electronic health records (EHRs).

**Objective:**

This study aimed to highlight that mining unstructured textual data with natural language processing techniques complements the available structured data and enables more comprehensive patient phenotyping. A proof-of-concept for patients diagnosed with specific autoimmune diseases is presented, in which the extraction of information on laboratory tests and drug treatments is performed.

**Methods:**

We collected EHRs available in the clinical data warehouse of the Greater Paris University Hospitals from 2012 to 2021 for patients hospitalized and diagnosed with 1 of 4 immune-mediated inflammatory diseases: systemic lupus erythematosus, systemic sclerosis, antiphospholipid syndrome, and Takayasu arteritis. Then, we built, trained, and validated natural language processing algorithms on 103 discharge summaries selected from the cohort and annotated by a clinician. Finally, all discharge summaries in the cohort were processed with the algorithms, and the extracted data on laboratory tests and drug treatments were compared with the structured data.

**Results:**

Named entity recognition followed by normalization yielded *F*_1_-scores of 71.1 (95% CI 63.6-77.8) for the laboratory tests and 89.3 (95% CI 85.9-91.6) for the drugs. Application of the algorithms to 18,604 EHRs increased the detection of antibody results and drug treatments. For instance, among patients in the systemic lupus erythematosus cohort with positive antinuclear antibodies, the rate increased from 18.34% (752/4102) to 71.87% (2949/4102), making the results more consistent with the literature.

**Conclusions:**

While challenges remain in standardizing laboratory tests, particularly with abbreviations, this work, based on secondary use of clinical data, demonstrates that automated processing of discharge summaries enriched the information available in structured data and facilitated more comprehensive patient profiling.

## Introduction

### Background

Since the 2010s, the widespread adoption of electronic health records (EHRs) and health data warehouses has enabled the development and application of new algorithms for patient phenotyping, which corresponds to the extraction of a set of observable patient characteristics, including laboratory test results, symptoms, diseases, and past or current treatments [1]. The automated extraction of these characteristics from large-scale databases supports predictive risk assessments, preselection for therapeutic trials, and pharmacovigilance analyses [2-4].

EHR data is typically categorized into 2 types: structured data and unstructured data. Structured data refers to directly queryable numerical values, such as laboratory test results or *International Classification of Diseases, Tenth Revision* (*ICD*-*10*) codes, while unstructured data encompasses raw clinical texts and medical imaging. Structured data from clinical warehouses is often incomplete, capturing only intrahospital records and excluding extrahospital information. For instance, a patient’s blood test conducted at an external laboratory before hospitalization might not be included. In addition, historical biological results in clinical databases are often limited to a few years. This is particularly problematic for conditions like autoimmune diseases, where historical immunologic results critical to the initial diagnosis are often documented only in clinical text rather than in structured data. Similarly, details about prior treatments are usually found only in textual records. Valuable information that is not present in structured data is often found in observations recorded in the discharge summaries [5]. The application of automated text analysis to this unstructured text, in conjunction with structured data, has already demonstrated increased effectiveness in predicting patients’ clinical courses [6-11].

Transforming unstructured data into structured formats involves multiple natural language processing (NLP) tasks. In this research, we primarily concentrate on named entity recognition (NER) and normalization, which are fundamental for extracting meaningful information from large volumes of unstructured clinical text.

NER refers to locating and classifying terms into predefined categories, such as drug name, laboratory test, or medical disorder. Traditional NER methods often depend on dictionary-based term-matching techniques, which require meticulously maintained lexical resources [12]. However, maintaining these resources can be both labor-intensive and error-prone. A more effective method treats NER as a sequence-labeling task using tagging systems like the beginning, inside, outside, unit, and last scheme, which is widely recognized in biomedical NER for its ease of implementation and efficiency [13,14]. Sequence labeling models, particularly conditional random fields [15], have been extensively used for NER. When combined with transformer-based architectures like bidirectional encoder representations from transformer (BERT), these models have set state-of-the-art performance benchmarks for NER in clinical and biomedical text analyses [16-19].

Following NER, the normalization process assigns standard codes (unique identifiers that correspond to concepts within established medical terminologies) to the detected terms. For example, standard codes, such as concept unique identifiers (CUIs) from the Unified Medical Language System (UMLS) [20], can be used to map detected entities like drug or laboratory tests to their corresponding concepts. Common normalization strategies often rely on exact or approximate string matching against predefined dictionaries. Tools, such as KnowledgeMap Concept Identifier [21], MetaMap [22], MedLEE [23], MedEx [24], HITEx [25], and cTAKES [26] have been widely adopted in phenotyping models [27-29]. The emergence of deep contextual embeddings, notably BERT [30], has revolutionized NLP methodologies, including normalization tasks. Current state-of-the-art approaches heavily use transformer-based encoders pretrained on domain-specific corpora, demonstrating substantial improvements in normalization [31-33].

Although large language models like GPT-4 [34] hold promise for biomedical applications, their current performance in tasks like NER and normalization remains limited [35]. Moreover, implementing these models at scale to extract phenotypes from large volumes of clinical documents poses considerable cost challenges.

### Goal of the Study

**Figure 1:**
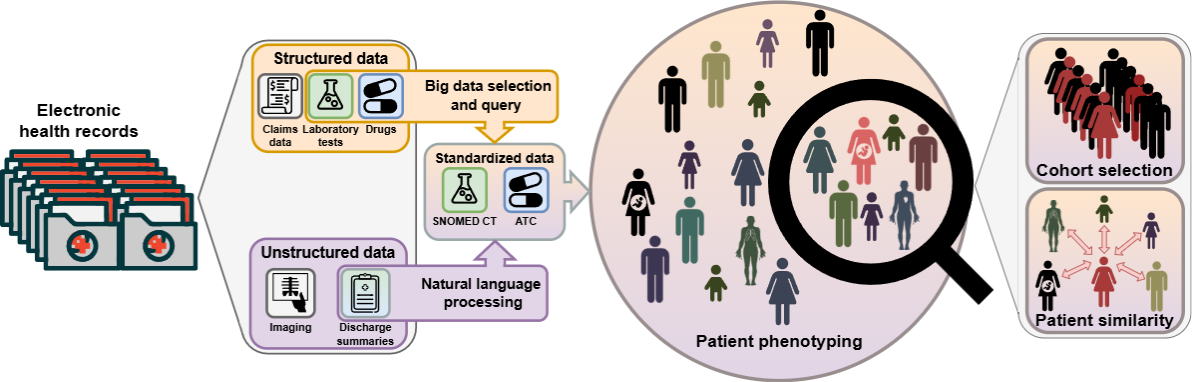
Overview of the end-to-end patient phenotyping pipeline. Structured and unstructured data are extracted from electronic health records, enabling large information retrieval, refining cohort selection, and facilitating more robust patient comparisons. ATC: Anatomical Therapeutic Chemical; SNOMED: Systematized Nomenclature of Medicine Clinical Terms.

The aim of the study was to provide a proof-of-concept for end-to-end patient phenotyping from their EHRs. Patient phenotyping refers to the process of characterizing patients based on their clinical features, such as clinical diagnoses, laboratory results, or drug treatments. Secondary uses of EHRs require the application of various processes to transform the data into meaningful variables. In this research, we focused specifically on leveraging discharge summaries (written in French) through NLP techniques to enrich the information contained in the structured data. We restricted our study to patients hospitalized for one of the following immune-mediated inflammatory diseases: systemic lupus erythematosus (SLE), systemic sclerosis, antiphospholipid syndrome (APS), and Takayasu arteritis (TA). As we analyzed autoimmune diseases, we also restricted phenotyping to the analysis of autoantibodies (laboratory tests) and immunosuppressive therapies (drugs), which are central to the management of these diseases. As shown in Figure 1, laboratory tests and drug therapies were extracted from both structured and unstructured data. Then, to analyze the data jointly, a standard concept code was assigned to each laboratory test using the Systematized Nomenclature of Medicine Clinical Terms (SNOMED CT; US edition) [36] and drug using the Anatomical Therapeutic Chemical (ATC) classification [37]. Our hypothesis was that incorporating the results of laboratory tests and drug treatments recorded in patients’ discharge summaries would complement the information available in structured data and enable more in-depth, interoperable phenotyping of patients, while remaining reliable.

## Methods

### Selected Diseases

As a proof of concept, we focused on 4 immune-mediated inflammatory diseases: SLE, systemic sclerosis, APS, and TA.

SLE is an autoimmune disease that mainly affects the skin, joints, and kidneys [38,39]. According to the revised 2019 EULAR/ACR classification criteria for SLE, patients are eligible for SLE criteria only if they have a positive antinuclear antibody ≥1/80 at least once. Anti-dsDNA and anti-Smith autoantibodies with high specificity for SLE are also included in the classification criteria for SLE. Therefore, we have chosen these 3 antibodies to identify SLE patients. Hydroxychloroquine, glucocorticoids, mycophenolate mofetil, cyclophosphamide, and belimumab are key treatments of SLE [40] and have been chosen to identify patients with SLE.

Systemic sclerosis is a rare autoimmune disease, inducing skin fibrosis, digestive disorders, such as gastroesophageal reflux disease and chronic pseudoocclusive syndrome, interstitial lung involvement, and sometimes inaugural renal crisis. Classification criteria are also based on specific autoantibodies, including anti-Scl-70, anticentromere, and anti-RNA polymerase III, which we have chosen to analyze here [41]. Therapeutic management is also based on glucocorticoids and immunosuppressive drugs, such as mycophenolate mofetil.

APS is a systemic autoimmune disease defined by the thrombosis or pregnancy morbidity in the presence of persistent antiphospholipid autoantibodies, lupus anticoagulant, IgG or IgM anticardiolipin, IgG or IgM anti-β2glycoprotein-1 antibodies. Treatment is based on curative anticoagulation with heparin, low-molecular-weight heparin, and antivitamin K [42].

TA is an inflammatory disease of the large arteries, leading to arterial stenosis in young people. C-reactive protein is used as an indicator of inflammation and disease activity in TA. Treatment is based on immunosuppressive therapies, such as glucocorticoids, and biologic, such as methotrexate or tocilizumab. Therefore, we have chosen to focus our analysis on these treatments.

Finally, in the context of the immunosuppressive treatments proposed, patients are at greater risk of infection; therefore, vaccination, particularly against pneumococcal and influenza infections, is recommended. Hence, we also looked for this information in the texts.

### Dataset Selection

The dataset used in this study comes from the clinical data warehouse (CDW) of the University Hospitals of Greater Paris (Assistance publique-hôpitaux de Paris; AP-HP). The CDW brings together information on all patients followed in the 39 teaching hospitals in the Paris region (>22,000 beds and 1.5 million hospitalizations per year) that use a common EHR software, ORBIS Dedalus Health care. This software has been gradually implemented in the 39 hospitals since 2012.

The dataset was extracted from the CDW research database, in the integrating biology and the bedside format [43]. The inclusion criteria for the study were as follows: all patients aged >15 years with SLE, systemic sclerosis, APS, or TA who had at least one stay at AP-HP hospitals initially from July 1, 2017, to December, 31, 2020. Patients in the database were selected in 2 ways: by the *ICD-10* codes of these 4 pathologies and by keywords present in the medical reports (using regular expression matching), as summarized in Table S1 in Multimedia Appendix 1 [20,32,33,36,37,44-51]. For these patients, the data available were demographic data; textual data, including all full-text medical reports, laboratory tests performed during patients’ stay, drug prescription, and administration when available; and medico-administrative coding data (*ICD-10*). The extraction covered all medical departments that could potentially manage patients with the 4 pathologies of interest: internal medicine and clinical immunology, nephrology, rheumatology, dermatology, pneumology, neurology, gastroenterology, oncology, hematology, infectious diseases, and emergency and intensive care.

**Figure 2:**
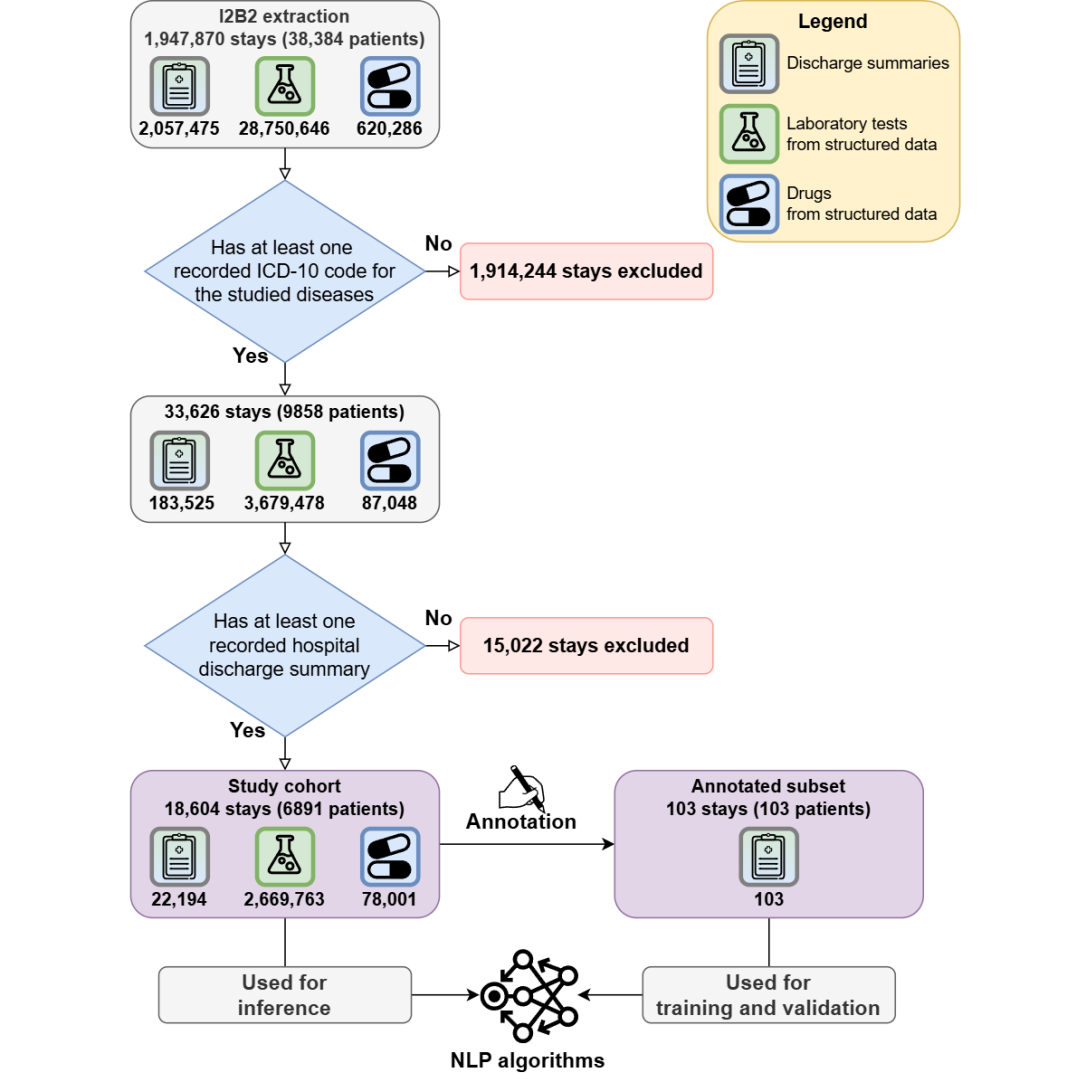
Cohort selection flowchart. Starting with integrating biology and bedside extraction of 1,947,870 stays (38,384 patients), the cohort was filtered to include stays with at least 1 International Classification of Diseases, 10th Revision (ICD-10) code corresponding to the studied diseases and at least 1 recorded hospital discharge summary. A final study cohort of 18,604 stays (6891 patients) was created, with 103 randomly selected discharge summaries annotated for training and validation purposes. NLP: natural language processing.

As this study involves the secondary use of real-life health data, from this large integrating biology and bedside extraction, we limited the study to EHRs with at least one *ICD-10* code corresponding to the diseases studied (SLE, systemic sclerosis, APS, or TA) and at least one recorded hospital discharge summary, as these are validated by a senior clinician. Subsequently, a subset of this study cohort of 103 hospital discharge summaries, each corresponding to a different patient, was randomly selected and annotated by a clinician (CG), following the same annotation rules as proposed by the national NLP clinical challenges 2022 [44]. Details regarding this annotation process are provided in the Annotation Guidelines section of Multimedia Appendix 1. The global approach of this work was to build, train, and validate NLP algorithms on the annotated subset before applying it to the full study cohort. Figure 2 presents the cohort selection process.

### End-to-End Pipeline

#### Overview

**Figure 3:**
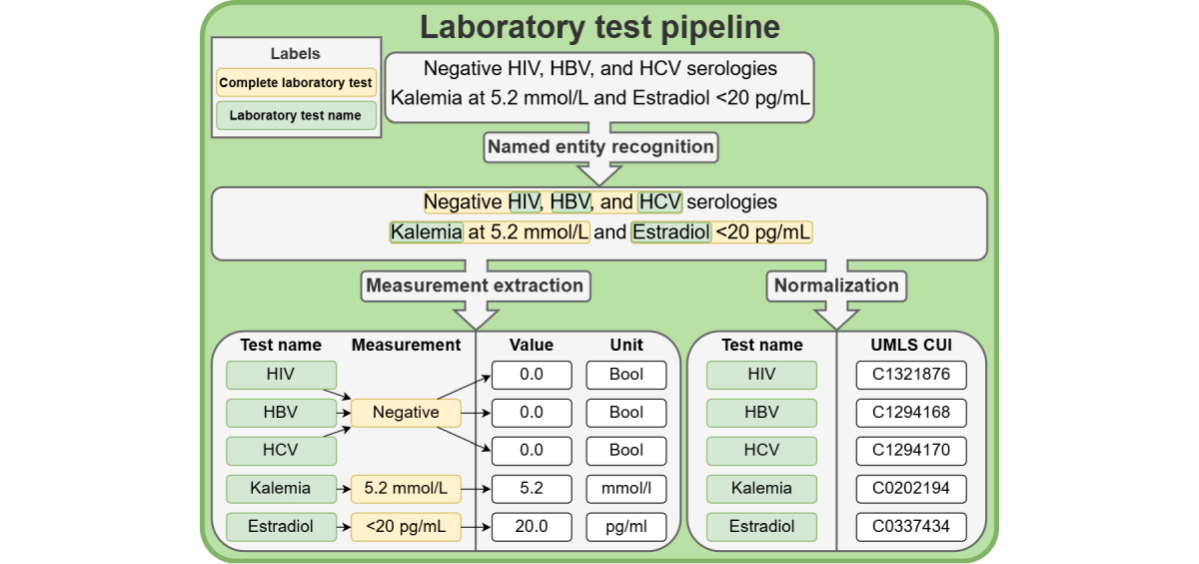
Diagram of the laboratory test pipeline. It takes raw text as input, which is processed by 3 algorithms in total. It starts with the extraction and classification of relevant terms into 2 categories: laboratory test name and complete laboratory test. Then, the measurements associated with the complete laboratory tests are extracted and standardized into 2 components: value and unit. Finally, the extracted laboratory test names are normalized to the concept unique identifiers (CUIs) of the Unified Medical Language System (UMLS). HBV: hepatitis B virus; HCV: hepatitis C virus.

**Figure 4:**
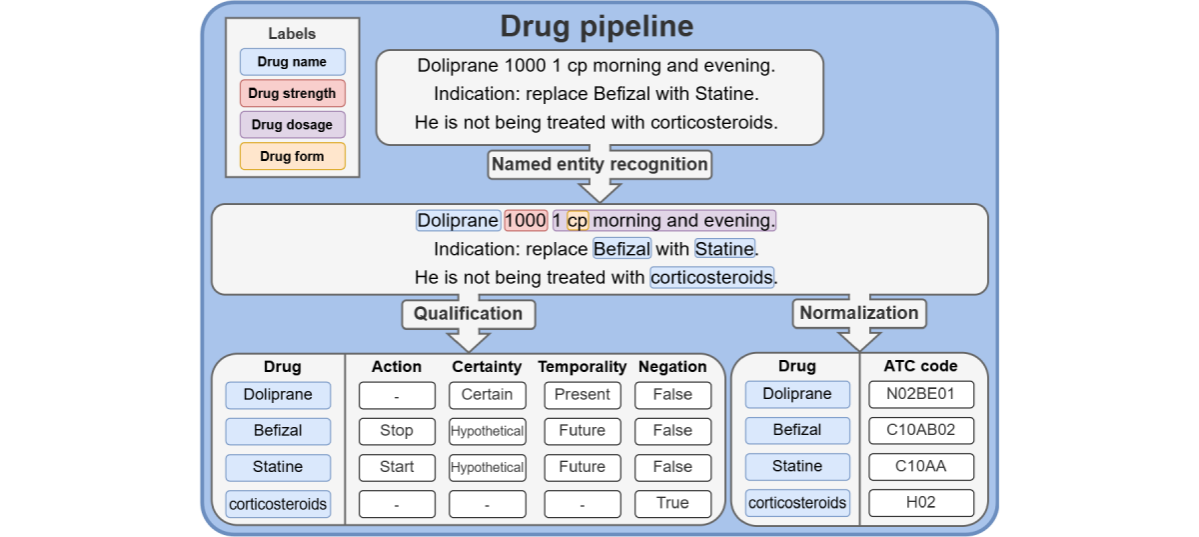
Diagram of the drug pipeline. It takes raw text as input, which is processed by 3 algorithms in total. It starts with the extraction and classification of relevant terms into 4 categories: name, strength, dosage, and form. At the same time, drugs are also qualified with several possible values: action (start, stop, increase, decrease, unique dose, and unknown), temporality (present, past, and future), certainty (certain, hypothetical, and conditional), and negation (true and false). Then, the extracted drugs are normalized according to the Anatomical Therapeutic Chemical (ATC) classification system. Cp: capsule.

The system presented in this work required 4 NLP tasks: (1) NER: this task identified and classified entities of interest mentioned in the text into predefined categories. The possible categories included drug name, drug strength, drug dosage, drug form, laboratory test name, and complete laboratory test. (2) Qualification: this task involved assigning predefined qualifiers to the recognized named entities. Only entities classified as “drug name” by the NER algorithm were qualified There were 4 qualifiers [52]: action (start, stop, increase, decrease, unique dose, and unknown), temporality (present, past, and future), certainty (certain, hypothetical, and conditional), as well as negation (true and false). (3) Measurement extraction: this task extracted and standardized the value and unit contained in the “complete laboratory test” entities detected by the NER algorithm. (4) Normalization: this task assigned predefined standard concepts to the recognized named entities. Each entity classified as “drug name” by the NER algorithm was assigned a code from the ATC classification system [37]. Each entity classified as “laboratory test name” by the NER algorithm was assigned a CUI of the UMLS [20] restricted to the laboratory procedure semantic type and the SNOMED CT US edition vocabulary [36]. As described in Figures 3 and Figure 4, the laboratory test pipeline and the drug pipeline involved both NER and normalization, while measurement extraction only concerned the laboratory test pipeline and qualification only concerned the drug pipeline.

All the work presented in this paper was programmed in Python. Tabular data were processed with Spark (version 2.4.8) and distributed over 160 central processing units in parallel. This computing process is scalable over a large amount of data. Then, the cohorts were analyzed using Pandas (version 1.3.5). Inference and training of the NLP algorithms have been achieved on a V100 graphics processing unit. The code developed to run the experiments is freely available in a GitHub repository: Aremaki/BioMedics [53]. The code makes extensive use of EDS-NLP (version 0.13.0) [54], a collaborative NLP framework that aims primarily at building hybrid multitask NLP pipelines and extracting information from French clinical notes. It has also been made publicly available under an open-source license (BSD 3-clause): aphp/edsnlp.

#### NER and Qualification Tasks

In the NER and qualification step, we experimented with different methods: a rule-based method as a preliminary approach, using the terms provided by the standard terminologies directly for exact matching. The ATC classification system [37] was used for drugs, and the SNOMED CT US edition vocabulary [36] was used for laboratory tests. A detailed description of the dictionaries is provided in Table S2 in Multimedia Appendix 1.

**Figure 5:**
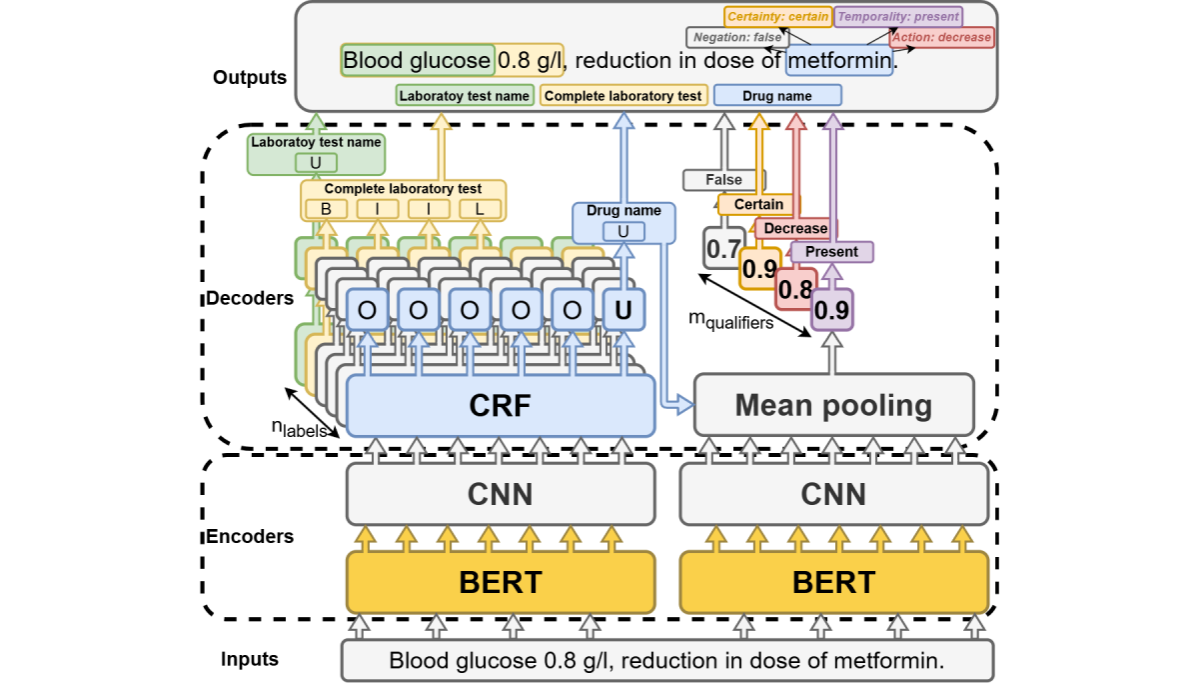
Diagram of the named entity recognition and qualification architecture for laboratory tests and drugs entities. BERT: bidirectional encoder representations from transformer; CNN: convolution neural network; CRF: conditional random field.

As a second approach, we experimented with a deep neural network architecture, described in Figure 5. The model consists of 2 BERTs encoders [30,55] followed by 2 convolution neural networks [56]. The first one is followed by a conditional random fields decoder [15] and a softmax block, which outputs probability vectors based on the beginning, inside, outside, unit, and last tagging scheme [14] to perform NER. The second is followed by a mean pooling layer and a softmax block to perform entity qualification. Several pretrained language models such as CamemBERT-EDS [45], CamemBERT-base [46], CamemBERT-bio [47], and DrBERT [48] have been compared.

To select the best approach, precision, recall, and *F*_1_-score were evaluated on 20 AP-HP discharge summaries. The performance of the rule-based method is presented in Table S3 in Multimedia Appendix 1, and the performance of the neural network method of each pretrained language model is presented in Tables S4 and S5 in Multimedia Appendix 1. The neural network approach using CamemBERT-EDS [45] was selected as the final model because it demonstrated superior performance compared to the other methods. Parameters of the architecture and fine-tuning are outlined in Table S6 in Multimedia Appendix 1.

#### Measurement Extraction Task

**Figure 6:**
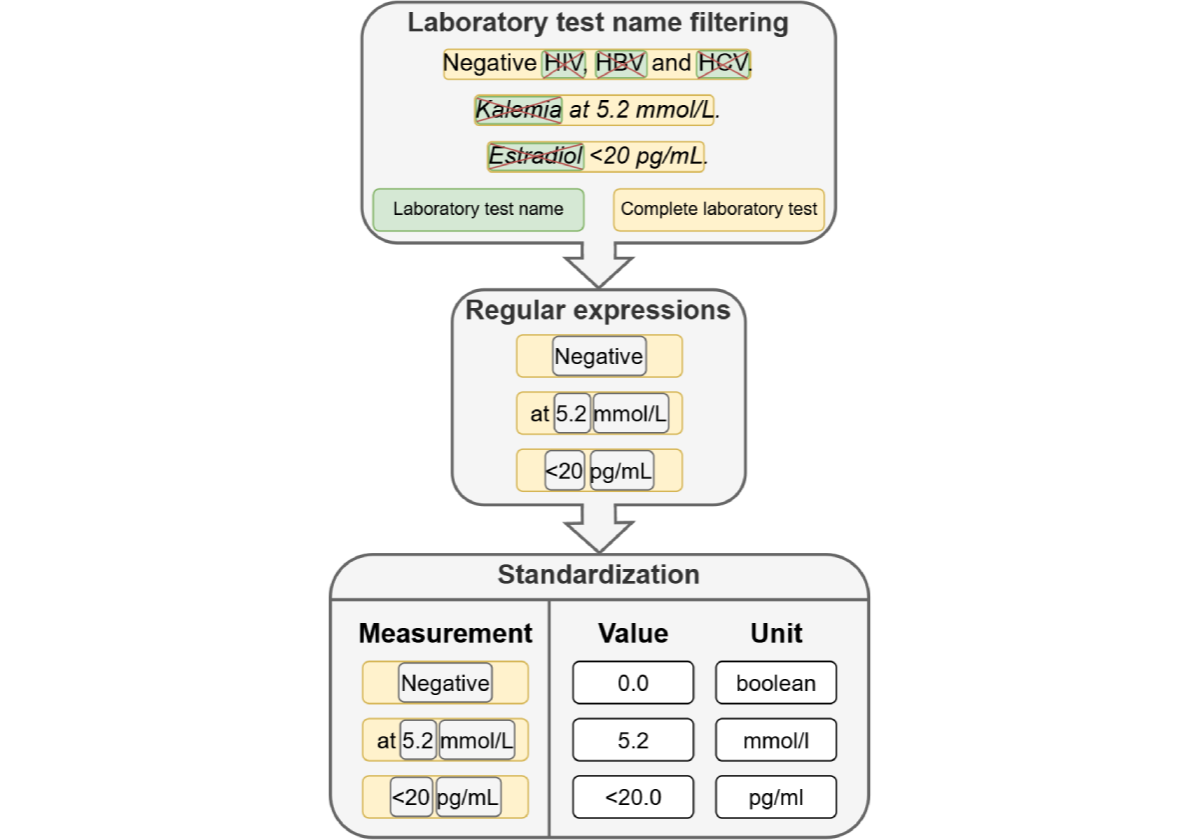
Diagram of the laboratory test measurement extraction process. HBV: hepatitis B virus; HCV: hepatitis C virus.

Extraction and standardization of the numerical value and unit were carried on the outputs of the NER step, which extracts the complete laboratory test entity from the text in a single block (laboratory test name, numerical value, and unit). The extraction and standardization were achieved with a rule-based algorithm using regular expressions. The algorithm steps are described in Figure 6: (1) the laboratory test names were removed from the complete laboratory test entity, (2) regular expressions were designed to extract the numerical or qualitative value and the unit, and (3) qualitative values (eg, “positive,” “negative,” or “normal”) were standardized into graded numbers (1.0, 0.0, or 0.5), while units were converted to conventional standards.

#### Normalization Task

**Figure 7:**
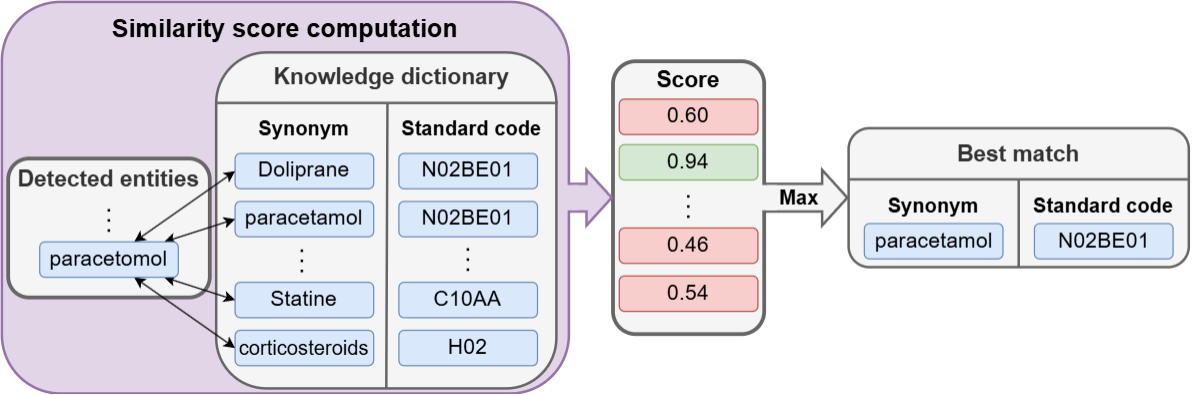
Diagram of the normalization process. In this example, the best match of “paracetomol,” written with a typo, is “paracetamol,” which provides the correct Anatomical Therapeutic Chemical (ATC) code.

The aim of the normalization step was to determine the standard code for each detected entity classified as “laboratory test name” and “drug name.” As described in Figure 7, for a given detected term, similarity scores were computed for all terms of a knowledge dictionary. The resulting standard code corresponded to the term with the highest similarity score. For drugs, the knowledge dictionary is an aggregation of 2 open-source dictionaries of drug names with their corresponding ATC codes: the UMLS [20], restricted to the French ATC vocabulary [37], and the Unique Drug Interoperability Repository created by the French National Agency for Medicines and Health Products Safety [57]. For laboratory tests, the knowledge dictionary consists of all the French and English synonyms of the UMLS [20] restricted to the laboratory procedure semantic type and the SNOMED CT US edition vocabulary [36]. We experimented with 2 types of score computation: (1) fuzzy matching methods that directly compared word characters: Jaro-Winkler Distance [49] and Levenshtein distance [50], as well as (2) neural network–based methods that compute cosine similarity scores between the embeddings of the words: CODER-all [33] and SapBERT-all [32]. Table S7 in Multimedia Appendix 1 presents the performance of each method. The neural-based method with CODER-all seems to be significantly better for laboratory tests. However, for drugs, there is no significantly better solution, so we used the Jaro-Winkler Distance [49] method because it is less computationally expensive.

### Ethical Considerations

The research protocol for this project was approved in 2020 by the institutional review board of Assistance Publique – Hôpitaux de Paris (AP-HP) (20-93). All data used in this study were collected as part of routine medical care, and their use for research purposes falls under the ethical guidelines of the institutional review board. All patient data were pseudonymized to ensure privacy and comply with data protection regulations. No financial compensation was provided, as the study relied solely on retrospective data from electronic health records.

## Results

### Dataset Description

The number of patients, hospitalizations, and discharge summaries for each disease of the study cohort are given in Table 1. The age distribution and the distribution of admission start dates for each disease are presented in Figure S1 in Multimedia Appendix 1.

**Table 1:** Data description of the study cohort. Number of patients, hospitalizations, and discharge summaries for each studied disease. The number of discharge summaries is higher than the number of hospitalizations, as patients may change departments several times during the same stay (eg, be transferred to an intensive care unit, etc).

Disease	Number of patients	Number of hospitalizations	Number of discharge summaries
Antiphospholipid syndrome	1059	1818	2380
Lupus	4102	10,445	12,500
Systemic sclerosis	2031	6455	7585
Takayasu arteritis	252	833	965

We performed various analyses on the extracted data combined with the structured data from the cohort. The results are reported in 2 sections. The first section presents the performance of the NLP algorithms on the annotated subset of 103 discharge summaries. The second section is about the application of these algorithms to the 22,194 discharge summaries included in the study cohort. It described the contribution of unstructured data to structured data.

### NLP Performance

#### Overview

The performance of the model was evaluated on 4 main tasks: NER, qualification, measurement extraction, and normalization. This evaluation was conducted using 2 datasets: 103 manually annotated discharge summaries from the study cohort and the publicly available Quaero FrenchMed corpus [51]. Performance metrics, including precision, recall, and F_1_-score, were calculated and reported along with 95% CIs derived using the empirical bootstrap method at the discharge summary level [58]. It is important to note that while NER and qualification required annotated data for both training and testing, measurement extraction and normalization only required annotated data for testing. Therefore, all 103 discharge summaries were annotated for NER and qualification, with 83 (80.6%) randomly selected for training and the remaining 20 (19.4%) used for testing. These 20 (19.4%) test documents were further annotated for measurement extraction. Of these 20 documents, 11 (10.7%) were additionally annotated for normalization, resulting in 668 annotated entities, which was a more time-consuming process.

#### NER Task

Our model was evaluated for the NER task on both the AP-HP annotated discharge summaries and the Quaero FrenchMed corpus [51]. The results of our annotated dataset containing 103 discharge summaries from AP-HP are presented in Table 2. Precision, recall, and *F*_1_-score were measured in a “strict” way, that is, a true positive result was obtained when a predicted entity and a gold entity had the exact same boundaries and label. The model achieved an overall *F*_1_-score of 88.8. The results on the Quaero FrenchMed corpus [51] are presented in Tables S8 and S9 in Multimedia Appendix 1. The model achieved an overall *F*_1_-score of 66.2 for MEDLINE corpus and 71.71 for the European medicines agency corpus.

**Table 2:** Performance of the model for named entity recognition on University Hospitals of Greater Paris (Assistance Publique-Hôpitaux de Paris) discharge summaries. The model was trained on 83 annotated discharge summaries and tested on 20 discharge summaries. Each result was bootstrapped by discharge summary to provide a 95% CI given inside the brackets.

Label	Number of entities (95% CI)	Precision (95% CI)	Recall (95% CI)	*F*_1_-score (95% CI)
Laboratory test name	1292 (1603-1010)	90.6 (88.5-92.7)	89.9 (87.5-92.2)	90.3 (88.2-92.3)
Complete laboratory test	1041 (1323-793)	86.2 (84.3-88.3)	83.6 (79.1-87.5)	84.9 (82.1-87.5)
Drug name	585 (731-454)	90.8 (85.8-95.4)	92.5 (88.4-95.7)	91.6 (87.5-95.1)
Drug dosage	276 (368-194)	89.1 (85.4-94.1)	86.2 (81.7-90.8)	87.7 (84.0-92.1)
Drug form	170 (247-106)	86.5 (78.8-93.4)	94.1 (91.5-97.6)	90.1 (85.6-94.0)
Drug strength	130 (196-77)	93.1 (87.7-97.0)	93.8 (89.2-97.6)	93.5 (89.3-96.6)
Overall	3494 (4194-2885)	89.1 (87.5-90.8)	88.5 (86.4-90.5)	88.8 (87.1-90.5)

#### Qualification Task

The model was evaluated for the qualification task and achieved an *F*_1_-score of 78.8 on our annotated dataset containing 103 discharge summaries from AP-HP. The results are presented in Table S10 in Multimedia Appendix 1. On the test set, the model achieved an overall *F*_1_-score of 78.8.

#### Measurement Extraction Task

The model was evaluated for the measurement extraction task on 20 annotated discharge summaries from AP-HP. Precision, recall, and *F*_1_-score are presented in Table S11 in Multimedia Appendix 1 and were measured in a “strict” way: a true positive result was obtained when a predicted measurement and a gold measurement had the same value and unit. The algorithm obtained an *F*_1_-score of 96.7.

#### Normalization Task

The rule-based algorithm for drug name normalization and the deep learning algorithm for the laboratory test name normalization were both evaluated on 11 annotated discharge summaries from AP-HP, 3 documents from European medicines agency, and 833 titles from MEDLINE [51]. For the evaluation, a true positive result was obtained when the predicted code of an entity was part of the list of annotated gold standard codes. Precision, recall, and *F*_1_-score are presented in Table S12 in Multimedia Appendix 1. On our dataset of discharge summaries, the rule-based method for drug names achieved an *F*_1_-score of 92.9 and the neural network–based method used for laboratory tests achieved an *F*_1_-score of 82.2.

#### End-to-End Pipeline

The model was evaluated on the NER and normalization task using 11 annotated discharge summaries from AP-HP. Precision, recall, and *F*_1_-score were measured in a “strict” way: a true positive result was obtained when a predicted entity and a gold entity had exactly the same boundaries and label and the predicted standard code of the entity was part of the list of annotated gold standard codes. Precision, recall, and *F*_1_-score are presented in Table 3. We obtained an *F*_1_-score of 71.1 for laboratory tests and 89.3 for drug names.

**Table 3:** Performance of the models for named entity recognition and normalization tasks on University Hospitals of Greater Paris (Assistance Publique-Hôpitaux de Paris) discharge summaries. The model was tested on 11 discharge summaries. Each result was bootstrapped by discharge summary to provide a 95% CI given inside the brackets.

Label	Number of entities (95% CI)	Precision (95% CI)	Recall (95% CI)	*F*_1_-score (95% CI)
Laboratory test name	356 (204-548)	72.0 (65.3-79.1)	70.2 (59.7-77.9)	71.1 (63.6-77.8)
Drug name	312 (206-424)	91.9 (88.9-93.5)	86.9 (82.9-90.0)	89.3 (85.9-91.6)

### Clinical Application

For each studied disease (SLE, systemic sclerosis, APS, and TA), each studied antibody, and each studied drug treatment, we reported the number of patients for whom we extracted a positive antibody or a prescribed drug treatment from both the structured and unstructured data of the study cohort. Lists of CUI codes for the studied antibodies and ATC codes for drug treatments are available in Tables S9 and S10 in Multimedia Appendix 1. We were particularly interested in the number of patients for whom we extracted positive antibodies or drug treatments from the unstructured data of their EHRs that were not available in the structured data. In the analysis of the unstructured data, only entities explicitly qualified as “certain” and not negated were retained.

#### Laboratory Test Results

Table 4 describes the number of antibody-positive patients for each disease, where positivity was determined either by test values exceeding reference ranges or by explicit interpretation of the test as positive. The first column shows the number of patients for whom we extracted at least 1 positive test from the structured data of their EHR. The second column shows the number of patients for whom we extracted at least 1 positive test from both the structured and unstructured data. The third column shows the number of patients for whom we extracted at least 1 positive test from the unstructured data, but only among patients without positive tests extracted from the structured data.

To ensure the reliability of the detected autoantibodies across the entire study cohort, we conducted a second evaluation focused specifically on the studied autoantibodies. While the precision was reported in Table 3 as 72% in the general case for all laboratory tests, this additional analysis aimed to ensure comparable performance for the autoantibodies studied. For this evaluation, we randomly selected 10 positively detected entities for each studied autoantibody, yielding a total of 110 entities. These entities, identified through NER, measurement extraction, and normalization, were reviewed by a clinician. The review identified 3 errors, resulting in a precision of 97.3%.

Finally, to identify potential errors made by the algorithm, we examined EHR cases in which a positive autoantibody appeared in the structured data but was not detected in the unstructured data. For each antibody, we randomly selected 10 EHRs (for some antibodies, fewer than 10 documents met this criterion, so we included all available cases). A clinician reviewed 63 discharge summaries from the EHRs and identified 2 types of algorithmic errors: (1) in 23 (26.5%) summaries, the algorithm either failed to detect the relevant antibody or did not normalize it correctly; and (2) in the remaining 40 (63.5%) summaries, either the antibody was not mentioned in the text, or the mention was interpreted as a negative result by the clinician.

**Table 4:** Number of patients with positive antibodies mentioned in the text and in structured data.

Disease and laboratory test		Number of patients with positive tests (ratio)		
		Structured data only, n (%)	Structured and unstructured data, n (%)	Benefits of the unstructured data, n (%)
**Antiphospholipid syndrome (1059 patients)**				
	Anticardiolipin antibody	184 (17.37)	478 (45.14)	294 (27.76)
	Anti-B2GP1 antibody	103 (9.73)	334 (31.54)	231 (21.81)
	Lupus anticoagulant	277 (26.16)	423 (39.94)	146 (13.79)
**Systemic lupus erythematosus (4102 patients)**				
	Antinuclear antibody	752 (18.33)	2949 (71.89)	2197 (53.56)
	Anti-DNA antibodies	541 (13.19)	2174 (53)	1633 (39.81)
	Anti-Smith antibodies	255 (6.22)	858 (20.92)	603 (14.7)
**Systemic sclerosis (2031 patients)**				
	Anti-RNA polymerase III antibody	39 (1.92)	156 (7.68)	117 (5.76)
	Anti-Scl-70 antibodies	97 (4.78)	568 (27.97)	471 (23.19)
	Anticentromere antibody	144 (7.09)	609 (29.99)	465 (22.9)

#### Drug Treatments

Table 5 describes the number of patients with drug treatments for each of the studied drugs related to the respective disease. The first column presents the number of patients for whom we extracted at least 1 drug treatment from the structured data of their EHR. The second column presents the number of patients for whom we extracted at least 1 drug treatment from both the structured and the unstructured data. The third column provides the number of patients for whom we extracted at least 1 drug treatment from the unstructured data but only among the patients without drug treatment extracted from the structured data.

**Table 5:** Number of patients with drug treatments. It describes the number of patients with drug treatments for each of the studied drugs related to the respective disease, extracted from structured and unstructured data. The proportions in percentage of the total number of patients diagnosed with the respective disease are given inside parenthesis.

Diseases and drugs		Number of patients with drug treatments (ratio)		
		Structured data only, n (%)	Structured and unstructured data, n (%)	Benefits of the unstructured data, n (%)
**Antiphospholipid syndrome (1059 patients)**				
	VKA	186 (17.56)	628 (59.3)	442 (41.74)
	Heparin	238 (22.47)	677 (63.94)	439 (41.46)
	Oral anticoagulant	47 (4.44)	177 (16.72)	130 (12.28)
**Systemic lupus erythematosus (4102 patients)**				
	Systemic glucocorticoids	950 (23.16)	3308 (80.64)	2358 (57.49)
	Cyclophosphamide	64 (1.56)	894 (21.79)	830 (20.23)
	Mycophenolate mofetil	301 (7.34)	1263 (30.78)	962 (23.46)
	Rituximab	75 (1.83)	709 (17.28)	634 (15.46)
	Belimumab	43 (1.05)	247 (6.02)	204 (4.97)
	Methotrexate	112 (2.73)	963 (23.48)	851 (20.75)
	Hydroxychloroquine	920 (22.43)	3520 (85.83)	2600 (63.4)
	Prevenar 13 vaccine	122 (2.97)	984 (23.99)	862 (21.02)
	Pneumovax vaccine	43 (1.05)	436 (10.63)	393 (9.58)
	Influenza vaccine	96 (2.34)	577 (14.07)	481 (11.73)
**Systemic sclerosis (2031 patients)**				
	Systemic glucocorticoids	258 (12.71)	1260 (62.06)	1002 (49.33)
	Cyclophosphamide	6 (0.3)	390 (19.2)	384 (18.91)
	Mycophenolate mofetil	88 (4.33)	463 (22.81)	375 (18.47)
	Rituximab	13 (0.64)	258 (12.71)	245 (12.07)
	Methotrexate	66 (3.25)	541 (26.63)	475 (23.39)
	Prevenar 13 vaccine	59 (2.9)	545 (26.84)	486 (23.93)
	Pneumovax vaccine	13 (0.64)	285 (14.03)	272 (13.4)
	Influenza vaccine	42 (2.07)	425 (20.93)	383 (18.87)
**Takayasu arteritis (252 patients)**				
	Systemic glucocorticoids	68 (27)	223 (88.49)	155 (61.51)
	Cyclophosphamide	0 (0)	18 (7.14)	18 (7.14)
	Tocilizumab	15 (5.95)	47 (18.65)	32 (12.7)
	Mycophenolate mofetil	9 (3.57)	21 (8.33)	12 (4.76)
	Rituximab	0 (0)	6 (2.38)	6 (2.38)
	Methotrexate	20 (7.94)	135 (53.57)	115 (45.63)
	Prevenar 13 vaccine	9 (3.57)	83 (32.94)	74 (29.37)
	Pneumovax vaccine	3 (1.19)	48 (19.05)	45 (17.86)
	Influenza vaccine	6 (2.38)	41 (16.27)	35 (13.89)

To ensure the precision of the drug treatments identified by the algorithm, we conducted a second evaluation specifically focused on the studied drugs. Although the general precision for all drugs had previously been reported as 91.9% in Table 3, this additional analysis aimed to confirm comparable performance for the specific drug treatments studied. For this evaluation, we randomly selected 10 positively detected entities per studied drug (spanning NER and normalization), resulting in a total of 130 entities. A clinician reviewed these entities individually and found no errors, corresponding to a precision of 100%.

#### Inference Time and Carbon Footprint

When considering scaling these methods to a CDW that may process hundreds of thousands of documents daily, it is critical to evaluate both processing speed and environmental impact. The entire NLP process on the 22,194 documents took 145 minutes on a graphics processing unit (Tesla V100-SXM2-32GB) and resulted in a total emission of 0.39 kg equivalent of CO_2_.

## Discussion

### Principal Findings

In this paper, we proposed a novel block-based algorithm for extracting and normalizing medical data from text, enabling fine-grained phenotyping of patients with autoimmune or autoinflammatory diseases. We demonstrated that these cascading algorithms significantly improve patient characterization compared to relying only on structured data. In addition, we provided detailed results for every step of the algorithm (NER, qualification, measurement extraction, and normalization), evaluated our method using a publicly available dataset, Quaero [51], and provided a comprehensive performance comparison between models.

Our work offers several strengths. Notably, we leveraged state-of-the-art language models, particularly the BERT model, for named entity extraction. Indeed, when compared with recent large language models, such as GPT, BERT models remain the most effective for the NER task [58]. We evaluated and compared several language models and various methods for each step, demonstrating strong performance outcomes. The model evaluated for the NER task on 20 annotated discharge summaries achieved high *F*_1_-scores: 90.3 for laboratory test names and 91.6 for drug names. Similarly, the model achieved high *F*_1_-scores for the qualification task, the measurement extraction task, and the overall end-to-end task. A posteriori precision analysis also showed very good results (97.3% for laboratory tests and 100% for drugs). Finally, as shown in Tables 4 and Table 5, our study highlights significant improvements in information availability by enriching structured data with information extracted from unstructured data.

Beyond these results, our findings are consistent with those of previous studies. For instance, 71.87% (2949/4102) of patients in the lupus cohort exhibited positive antinuclear antibodies (≥1/80), a finding that aligns with the clinical criteria for the disease [40]. Similarly, when compared with previous data [38,40], 85.81% (3520/4102) of patients with SLE were treated with hydroxychloroquine, and 80.64% (3308/4102) received corticosteroids during hospitalization. For comparison, a recent conference abstract by Eviatar et al [59] reported that 81% of patients were treated with hydroxychloroquine, 65% with systemic corticosteroids, and 55% with immunosuppressants (2259/4102, 55.07% in our study). In addition, 64.4% (682/1059) of patients with APS had at least one positive antibody assay. For patients with TA, the treatments were consistent with national recommendations [60], with 88.5% (223/252) of patients receiving systemic corticosteroids and 18.7% (47/252) treated with tocilizumab.

The clinical implications of algorithms that enable accurate patient phenotyping are substantial. They facilitate more precise recruitment of patients for studies, particularly therapeutic trials, and support clinical practice by addressing key questions, such as, “What happened to a patient like mine?” Prototypes are currently under development to construct cohorts of patients with similar characteristics to a specific individual under care, using information extracted from hospital reports. The algorithm we present can identify patients with comparable immune profiles (eg, matching positive antibodies) and analyze the treatments they received, offering valuable insights for personalized care.

### Limitations

However, there are several limitations to our study. A significant limitation lies in the complexity of standardizing laboratory tests, especially for tests with abbreviated terms. For instance, the glomerular filtration rate (or “DFG” in French) is not directly classified as a biological test in the SNOMED CT US edition [36], making it challenging to standardize. Similarly, the abbreviation “ACC” for lupus circulating anticoagulant is missing in the UMLS [20], which makes normalization difficult and partially explains the lower contribution of text-based analysis for this assay. In general, drug names are often written in a relatively standardized format in texts (using either trade names or generics), whereas the terminology for describing biological data tends to be more varied. For example, a clinician might describe “hemoglobin” using variations, such as “anemia at 9g/dL,” “Hb=9g/dL,” or “hemoglobin at 9,” among others. This variability complicates the normalization process for laboratory tests, leading to poorer performance compared with that of drug treatments. Another limitation is the relatively small evaluation sample size. Our NLP end-to-end system was evaluated on only 11 annotated clinical documents, comprising 668 annotated entities. This limited dataset is a consequence of the labor-intensive process involved in manually annotating CUIs and ATC codes, which constrained the number of documents we could feasibly annotate. Also, interannotator agreement could not be computed due to having a single annotator involved in the annotation process. To minimize potential biases, several precautions were taken. First, an expert clinician performed the annotations following strict guidelines, while the model was independently designed by a separate researcher. Second, the training and test datasets were created using distinct discharge summaries from different patients. These precautions reduce the risk of information leakage during model evaluation.

Finally, it is important to note that this study relies on the secondary use of “real-life” health care data. While clinical texts are central to characterizing patients, as demonstrated, they do not comprehensively capture all patient characteristics. Our error analysis revealed that for patients with both textual information and biological test results from the same hospitalization, 63% (40/63) of the biological tests were either not mentioned in the text or were interpreted by the clinician as negative results. To enhance the accuracy of patient phenotyping, we believe it is essential to incorporate both structured and textual data.

### Future Works

Moreover, we acknowledge that the analyses presented here are preliminary for each pathology, and we anticipate more detailed future work in this area. Particularly, it will be necessary to establish a precise relationship between target organ damage and antibody positivity, some of which are known to be more specific for certain types of damage. For example, anti-RNA polymerase III antibodies are more often associated with sclerodermic renal crisis [61], and triple positivity of APS antibodies is also a poor prognostic marker. The type and severity of organ damage should also be considered in conjunction with treatment options. These analyses will also be based on our current patient phenotyping work [62]. Analysis of the dosages associated with each treatment is not currently explored either, but work is in progress for this future step. Another direction is adapting our methodology to other languages. While the current implementation is tailored for French, the approach can be generalized by substituting the pretrained clinical BERT model with other language-specific alternatives, such as models pretrained for Spanish [63] or English [64]. However, successful adaptation would require annotated datasets specific to the new language, as well as adjustments to the terminology and clinical standards used in the target CDW. Beyond linguistic adaptability, the methods described could also be extended to unstructured data in different formats, such as imaging. Addressing these directions could advance this research toward a more comprehensive, multilingual, and multiformat phenotyping framework.

### Conclusions

To the best of our knowledge, this is the first study to automatically analyze such a large volume of patients with autoimmune diseases using data derived directly from text. It seems to us that this finer, text-based characterization of patients in the context of rare diseases could enable researchers to target them more effectively, and clinicians to bring synthesis to their management.

## Appendix

Multimedia Appendix 1 Annotation guidelines, supplementary tables, and figure.

## Abbreviations

AP-HP: University Hospitals of Greater Paris (Assistance publique-hôpitaux de Paris)
APS: antiphospholipid syndrome
ATC: Anatomical Therapeutic Chemical
BERT: bidirectional encoder representations from transformer
CDW: clinical data warehouse
CUI: concept unique identifier
EHR: electronic health record
ICD-10: International Classification of Diseases, Tenth Revision
NER: named entity recognition
NLP: natural language processing
SLE: systemic lupus erythematosus
SNOMED CT: Systematized Nomenclature of Medicine Clinical Terms
TA: Takayasu arteritis
UMLS: Unified Medical Language System

## Data Availability

The datasets generated or analyzed during this study are not publicly available due to their classification as sensitive data under the General Data Protection Regulation and the National Data Protection Commission. Access to the data requires prior approval from the local institutional review board and must follow the process outlined on its website [65]. In the case of non-University Hospitals of Greater Paris (Assistance Publique-Hôpitaux de Paris) researchers, the signature of a collaboration contract is mandatory.

## References

[1] Richesson RL, Hammond WE, Nahm M, Wixted D, Simon GE, Robinson JG, Bauck AE, Cifelli D, Smerek MM, Dickerson J, Laws RL, Madigan RA, Rusincovitch SA, Kluchar C, Califf RM (2013). Electronic health records based phenotyping in next-generation clinical trials: a perspective from the NIH Health Care Systems Collaboratory. J Am Med Inform Assoc.

[2] Gombar S, Callahan A, Califf R, Harrington R, Shah NH (2019). It is time to learn from patients like mine. NPJ Digit Med.

[3] Callahan A, Polony V, Posada JD, Banda JM, Gombar S, Shah NH (2021). ACE: the Advanced Cohort Engine for searching longitudinal patient records. J Am Med Inform Assoc.

[4] Frankovich J, Longhurst CA, Sutherland SM (2011). Evidence-based medicine in the EMR era. N Engl J Med.

[5] Zheng C, Ackerson B, Qiu S, Sy LS, Daily LI, Song J, Qian L, Luo Y, Ku JH, Cheng Y, Wu J, Tseng HF (2024). Natural language processing versus diagnosis code-based methods for postherpetic neuralgia identification: algorithm development and validation. JMIR Med Inform.

[6] Elkin PL, Mullin S, Mardekian J, Crowner C, Sakilay S, Sinha S, Brady G, Wright M, Nolen K, Trainer J, Koppel R, Schlegel D, Kaushik S, Zhao J, Song B, Anand E (2021). Using artificial intelligence with natural language processing to combine electronic health record's structured and free text data to identify nonvalvular atrial fibrillation to decrease strokes and death: evaluation and case-control study. J Med Internet Res.

[7] Seinen TM, Fridgeirsson EA, Ioannou S, Jeannetot D, John LH, Kors JA, Markus AF, Pera V, Rekkas A, Williams RD, Yang C, van Mulligen EM, Rijnbeek P (2022). Use of unstructured text in prognostic clinical prediction models: a systematic review. J Am Med Inform Assoc.

[8] Khurshid S, Reeder C, Harrington LX, Singh P, Sarma G, Friedman SF, Di Achille P, Diamant N, Cunningham JW, Turner AC, Lau ES, Haimovich JS, Al-Alusi MA, Wang X, Klarqvist MD, Ashburner JM, Diedrich C, Ghadessi M, Mielke J, Eilken HM, McElhinney A, Derix A, Atlas SJ, Ellinor PT, Philippakis AA, Anderson CD, Ho JE, Batra P, Lubitz SA (2022). Cohort design and natural language processing to reduce bias in electronic health records research. NPJ Digit Med.

[9] Idnay B, Zhang G, Chen F, Ta CN, Schelke MW, Marder K, Weng C (2025). Mini-mental status examination phenotyping for Alzheimer's disease patients using both structured and narrative electronic health record features. J Am Med Inform Assoc.

[10] Fraile Navarro D, Ijaz K, Rezazadegan D, Rahimi-Ardabili H, Dras M, Coiera E, Berkovsky S (2023). Clinical named entity recognition and relation extraction using natural language processing of medical free text: a systematic review. Int J Med Inform.

[11] Moqurrab SA, Ayub U, Anjum A, Asghar S, Srivastava G (2021). An accurate deep learning model for clinical entity recognition from clinical notes. IEEE J Biomed Health Inform.

[12] Mikheev A, Moens M, Grover C (1999). Named Entity recognition without gazetteers. Proceedings of the 9th conference on European chapter of the Association for Computational Linguistics.

[13] Ramshaw LA, Marcus MP, Armstrong S, Church K, Isabelle P, Manzi S, Tzoukermann E, Yarowsky D (1995). Text chunking using transformation-based learning. Natural Language Processing Using Very Large Corpora.

[14] Ratinov L, Roth D (2009). Design challenges and misconceptions in named entity recognition. Proceedings of the 13th Conference on Computational Natural Language Learning.

[15] Lafferty JD, McCallum A, Pereira FC (2001). Conditional random fields: probabilistic models for segmenting and labeling sequence data. Proceedings of the 18th International Conference on Machine Learning.

[16] Lample G, Ballesteros M, Subramanian S, Kawakami K, Dyer C (2016). Neural architectures for named entity recognition. Proceedings of the 2016 Conference of the North American Chapter of the Association for Computational Linguistics: Human Language Technologies.

[17] Sung M, Jeong M, Choi Y, Kim D, Lee J, Kang J (2022). BERN2: an advanced neural biomedical named entity recognition and normalization tool. Bioinformatics.

[18] Jonker RA, Almeida T, Antunes R, Almeida JR, Matos S (2024). Multi-head CRF classifier for biomedical multi-class named entity recognition on Spanish clinical notes. Database (Oxford).

[19] Cardon R, Grabar N, Grouin C, Hamon T (2020). Presentation of the’assessment campaign DEFT 2020: textual similarity in open domain and extraction of’accurate information in clinical cases (presentation of the DEFT 2020 challenge : open domain textual similarity and precise information extraction from clinical cases). Proceedings of the 6th joint conference Days of Studies on the Word (JEP, 33rd edition), Automatic Processing of Natural Languages (TALN, 27th edition), Meeting of Research Students in Computer Science for Automatic Language Processing (RECITAL, 22nd edition). Workshop Defi Fouille de Textes.

[20] Bodenreider O (2004). The Unified Medical Language System (UMLS): integrating biomedical terminology. Nucleic Acids Res.

[21] Denny JC, Irani PR, Wehbe FH, Smithers JD, Spickard A (2003). The KnowledgeMap project: development of a concept-based medical school curriculum database. AMIA Annu Symp Proc.

[22] Aronson AR, Lang FM (2010). An overview of MetaMap: historical perspective and recent advances. J Am Med Inform Assoc.

[23] Friedman C, Shagina L, Socratous SA, Zeng X (1996). A WEB-based version of MedLEE: a medical language extraction and encoding system. Proc AMIA Annu Fall Symp.

[24] Xu H, Stenner SP, Doan S, Johnson KB, Waitman LR, Denny JC (2010). MedEx: a medication information extraction system for clinical narratives. J Am Med Inform Assoc.

[25] Zeng QT, Goryachev S, Weiss S, Sordo M, Murphy SN, Lazarus R (2006). Extracting principal diagnosis, co-morbidity and smoking status for asthma research: evaluation of a natural language processing system. BMC Med Inform Decis Mak.

[26] Savova GK, Masanz JJ, Ogren PV, Zheng J, Sohn S, Kipper-Schuler KC, Chute CG (2010). Mayo clinical Text Analysis and Knowledge Extraction System (cTAKES): architecture, component evaluation and applications. J Am Med Inform Assoc.

[27] Bejan CA, Xia F, Vanderwende L, Wurfel MM, Yetisgen-Yildiz M (2012). Pneumonia identification using statistical feature selection. J Am Med Inform Assoc.

[28] Liao KP, Cai T, Gainer V, Goryachev S, Zeng-treitler Q, Raychaudhuri S, Szolovits P, Churchill S, Murphy S, Kohane I, Karlson EW, Plenge RM (2010). Electronic medical records for discovery research in rheumatoid arthritis. Arthritis Care Res (Hoboken).

[29] Carroll RJ, Eyler AE, Denny JC (2011). Naïve electronic health record phenotype identification for rheumatoid arthritis. AMIA Annu Symp Proc.

[30] Devlin J, Chang MW, Lee K, Toutanova K (2019). BERT: pre-training of deep bidirectional transformers for language understanding. Proceedings of the 2019 Conference of the North American Chapter of the Association for Computational Linguistics: Human Language Technologies.

[31] French E, McInnes BT (2023). An overview of biomedical entity linking throughout the years. J Biomed Inform.

[32] Liu F, Shareghi E, Meng Z, Basaldella M, Collier N (2021). Self-alignment pretraining for biomedical entity representations. Proceedings of the 2021 Conference of the North American Chapter of the Association for Computational Linguistics: Human Language Technologies.

[33] Yuan Z, Zhao Z, Sun H, Li J, Wang F, Yu S (2022). CODER: knowledge-infused cross-lingual medical term embedding for term normalization. J Biomed Inform.

[34] Achiam OJ, Adler S, Agarwal S, Ahmad L, Akkaya I, Aleman FL, Almeida D, Altenschmidt J, Altman S, Anadkat S, Avila R, Babuschkin I, Balaji S, Balcom V, Baltescu P, Bao H, Bavarian M, Belgum J, Bello I, Berdine J, Bernadett-Shapiro G, Berner C, Bogdonoff L, Boiko O, Boyd M, Brakman A, Brockman G, Brooks T, Brundage M, Button K, Cai T, Campbell R, Cann A, Carey B, Carlson C, Carmichael R, Chan B, Chang C, Chantzis F, Chen D, Chen S, Chen R, Chen J, Chen M, Chess B, Cho C, Chu C, Chung H, Cummings D, Currier J, Dai Y, Decareaux C, Degry T, Deutsch N, Deville D, Dhar A, Dohan D, Dowling S, Dunning S, Ecoffet A, Eleti A, Eloundou T, Farhi D, Fedus L, Felix N, Fishman S, Forte J, Fulford I, Gao L, Georges E, Gibson C, Goel V, Gogineni T, Goh G, Gontijo-Lopes R, Gordon J, Grafstein M, Gray S, Greene R, Gross J, Gu S, Guo Y, Hallacy C, Han J, Harris J, He Y, Heaton M, Heidecke J, Hesse C, Hickey A, Hickey W, Hoeschele P, Houghton B, Hsu K, Hu S, Hu X, Huizinga J, Jain S, Jain S, Jang J, Jiang A, Jiang R, Jin H, Jin D, Jomoto S, Jonn B, Jun H, Kaftan T, Kaiser L, Kamali A, Kanitscheider I, Keskar N, Khan T, Kilpatrick L, Kim J, Kim C, Kim Y, Kirchner H, Kiros J, Knight M, Kokotajlo D, Kondraciuk L, Kondrich A, Konstantinidis A, Kosic K, Krueger G, Kuo V, Lampe M, Lan I, Lee T, Leike J, Leung J, Levy D, Li C, Lim R, Lin M, Lin S, Litwin M, Lopez T, Lowe R, Lue P, Makanju A, Malfacini K, Manning S, Markov T, Markovski Y, Martin B, Mayer K, Mayne A, McGrew B, McKinney S, McLeavey C, McMillan P, McNeil J, Medina D, Mehta A, Menick J, Metz L, Mishchenko A, Mishkin P, Monaco V, Morikawa E, Mossing D, Mu T, Murati M, Murk O, M?ely D, Nair A, Nakano R, Nayak R, Neelakantan A, Ngo R, Noh H, Long O, O?Keefe C, Pachocki J, Paino A, Palermo J, Pantuliano A, Parascandolo G, Parish J, Parparita E, Passos A, Pavlov M, Peng A, Perelman A, Peres FD, Petrov M, Pinto HD, Pokorny M, Pokrass M, Pong V, Powell T, Power A, Power B, Proehl E, Puri R, Radford A, Rae J, Ramesh A, Raymond C, Real F, Rimbach K, Ross C, Rotsted B, Roussez H, Ryder N, Saltarelli M, Sanders T, Santurkar S, Sastry G, Schmidt H, Schnurr D, Schulman J, Selsam D, Sheppard K, Sherbakov T, Shieh J, Shoker S, Shyam P, Sidor S, Sigler E, Simens M, Sitkin J, Slama K, Sohl I, Sokolowsky B, Song Y, Staudacher N, Such F, Summers N, Sutskever I, Tang J, Tezak N, Thompson M, Tillet P, Tootoonchian A, Tseng E, Tuggle P, Turley N, Tworek J, Uribe J, Vallone A, Vijayvergiya A, Voss C, Wainwright C, Wang J, Wang A, Wang B, Ward J, Wei J, Weinmann C, Welihinda A, Welinder P, Weng J, Weng L, Wiethoff M, Willner D, Winter C, Wolrich S, Wong H, Workman L, Wu S, Wu J, Wu M, Xiao K, Xu T, Yoo S, Yu K, Yuan Q, Zaremba W, Zellers R, Zhang C, Zhang M, Zhao S, Zheng T, Zhuang J, Zhuk W, Zoph B (2024). GPT-4 technical report. arXiv. Preprint posted online March 4, 2024.

[35] Tian S, Jin Q, Yeganova L, Lai P, Zhu Q, Chen X, Yang Y, Chen Q, Kim W, Comeau DC, Islamaj R, Kapoor A, Gao X, Lu Z (2023). Opportunities and challenges for ChatGPT and large language models in biomedicine and health. Brief Bioinform.

[36] SNOMED CT. US National Library of Medicine.

[37] Anatomical therapeutic chemical (ATC) classification. World Health Organization.

[38] Fanouriakis A, Kostopoulou M, Alunno A, Aringer M, Bajema I, Boletis JN, Cervera R, Doria A, Gordon C, Govoni M, Houssiau F, Jayne D, Kouloumas M, Kuhn A, Larsen JL, Lerstrøm K, Moroni G, Mosca M, Schneider M, Smolen JS, Svenungsson E, Tesar V, Tincani A, Troldborg A, van Vollenhoven R, Wenzel J, Bertsias G, Boumpas DT (2019). 2019 update of the EULAR recommendations for the management of systemic lupus erythematosus. Ann Rheum Dis.

[39] Aringer M, Costenbader K, Daikh D, Brinks R, Mosca M, Ramsey-Goldman R, Smolen JS, Wofsy D, Boumpas DT, Kamen DL, Jayne D, Cervera R, Costedoat-Chalumeau N, Diamond B, Gladman DD, Hahn B, Hiepe F, Jacobsen S, Khanna D, Lerstrøm K, Massarotti E, McCune J, Ruiz-Irastorza G, Sanchez-Guerrero J, Schneider M, Urowitz M, Bertsias G, Hoyer BF, Leuchten N, Tani C, Tedeschi SK, Touma Z, Schmajuk G, Anic B, Assan F, Chan TM, Clarke AE, Crow MK, Czirják L, Doria A, Graninger W, Halda-Kiss B, Hasni S, Izmirly PM, Jung M, Kumánovics G, Mariette X, Padjen I, Pego-Reigosa JM, Romero-Diaz J, Rúa-Figueroa Fernández Í, Seror R, Stummvoll GH, Tanaka Y, Tektonidou MG, Vasconcelos C, Vital EM, Wallace DJ, Yavuz S, Meroni PL, Fritzler MJ, Naden R, Dörner T, Johnson SR (2019). 2019 European League against rheumatism/American College of Rheumatology Classification Criteria for Systemic Lupus Erythematosus. Arthritis Rheumatol.

[40] Lupus Systémique de l'adulte et de l'enfant. Haute Autorité de Santé.

[41] (2018). Sclérodermie Systémique. Haute Autorité de Santé.

[42] Syndrome des Anti-Phospholipides de l’adulte et de l’enfant. Haute Autorité de Santé.

[43] i2b2: informatics for integrating biology and the bedside. i2b2.

[44] Mahajan D, Liang JJ, Tsou C, Uzuner Ö (2023). Overview of the 2022 n2c2 shared task on contextualized medication event extraction in clinical notes. J Biomed Inform.

[45] Dura B, Jean C, Tannier X, Calliger A, Bey R, Neuraz A, Flicoteaux R (2022). Learning structures of the French clinical language:development and validation of word embedding models using 21 million clinical reports from electronic health records. arXiv. Preprint posted online July 26, 2022.

[46] Martin L, Muller B, Suárez PJ, Dupont Y, Romary L, de la Clergerie ÉV, Seddah D, Sagot B (2020). CamemBERT: a tasty French language model. Proceedings of the 58th Annual Meeting of the Association for Computational Linguistics.

[47] Touchent R, Romary L, de La Clergerie É (2024). CamemBERT-bio: leveraging continual pre-training for cost-effective models on French biomedical data. Proceedings of the 2024 Joint International Conference on Computational Linguistics, Language Resources and Evaluation.

[48] Labrak Y, Bazoge A, Dufour R, Rouvier M, Morin E, Daille B, Gourraud PA (2023). DrBERT: a robust pre-trained model in French for biomedical and clinical domains. Proceedings of the 61st Annual Meeting of the Association for Computational Linguistics.

[49] Winkler WE (2022). String comparator metrics and enhanced decision rules in the Fellegi-Sunter model of record linkage. Bureau of the Census.

[50] Levenshtein VI (1965). Binary codes capable of correcting deletions, insertions, and reversals. Sov Phys Dokl.

[51] Grouin C, Leixa J, Névéol A, Rosset S, Tannier X, Zweigenbaum P The Quaero French Medical Corpus: a ressource for medical entity recognition and normalization. paperswithcode.

[52] Mahajan D, Liang JJ, Tsou CH (2021). Toward understanding clinical context of medication change events in clinical narratives. AMIA Annu Symp Proc.

[53] Remaki A (2022). BioMedics. Zenodo.

[54] Wajsburt P, Petit-Jean T, Dura B, Cohen A, Jean C, Bey R EDS-NLP: efficient information extraction from French clinical notes. zenodo.

[55] Vaswani A, Shazeer N, Parmar N, Uszkoreit J, Jones L, Gomez A, Kaiser Ł, Polosukhin I (2017). Attention is all you need. Proceedings of the 31st International Conference on Neural Information Processing Systems.

[56] Abas AR, Elhenawy I, Zidan M, Othman M (2021). BERT-CNN: a deep learning model for detecting emotions from text. Comput Mater Contin.

[57] Catalogue des terminologies. Ministère du Travail, de la Santé et des Solidarités & ANS.

[58] Dekking FM, Kraaikamp C, Lopuhaä HP, Meester LE, Dekking FM, Kraaikamp C, Lopuhaä HP, Meester LE (2005). The bootstrap. A Modern Introduction to Probability and Statistics: Understanding Why and How.

[59] Eviatar T, Yahalom R, Livnat I, Elboim M, Elkayam O, Chodick G, Rosenberg V, Paran D (2024). Real-world treatment patterns in patients with systemic lupus erythematosus: associations with comorbidities and damage. Lupus Sci Med.

[60] Artérite de Takayasu. Haute Autorité de Santé.

[61] Mouthon L, Bussone G, Berezné A, Noël LH, Guillevin L (2014). Scleroderma renal crisis. J Rheumatol.

[62] Gérardin C, Mageau A, Mékinian A, Tannier X, Carrat F (2022). Construction of cohorts of similar patients from automatic extraction of medical concepts: phenotype extraction study. JMIR Med Inform.

[63] Carrino CP, Llop J, Pàmies M, Gutiérrez-Fandiño A, Armengol-Estapé J, Silveira-Ocampo J, Valencia A, Gonzalez-Agirre A, Villegas M (2022). Pretrained biomedical language models for clinical NLP in Spanish. Proceedings of the 21st Workshop on Biomedical Language Processing.

[64] Lee J, Yoon W, Kim S, Kim D, Kim S, So C, Kang J (2020). BioBERT: a pre-trained biomedical language representation model for biomedical text mining. Bioinformatics.

[65] Entrepôt de Données de Santé. Assistance Hoptaux Publique de Paris.

