# Supplementary material for: Improving Phenotyping of Patients With Immune-Mediated Inflammatory Diseases Through Automated Processing of Discharge Summaries: Multicenter Cohort Study
Source: JMIR Med Inform. 2025 Apr 9;13:e68704. doi: 10.2196/68704 (PMC12018854; doi:10.2196/68704)
Supplement: Multimedia Appendix 1 [file medinform_v13i1e68704_app1.docx]

Supplementary materials

# **ICD-10 CODES FOR DIAGNOSES**

| **Pathologies** | **ICD-10 codes** | **Keywords in discharge summaries** | **No. of corresponding patients** |
| --- | --- | --- | --- |
| **Antiphospholipid syndrome** | D686 | "SAPL", "anti-phospholipid syndrome", "CAPS” | 16 401 |
| **Systemic lupus erythematosus** | M310, M321, M328, M329, L930, L931 | "lupus” | 22 252 |
| **Systemic sclerosis** | M340, M341, M348, M349 | "systemic sclerosis”, “CREST” | 7 711 |
| **Takayasu’s arteritis** | M314 | "takayasu” | 576 |

**Table S1.** Queries input used to extract the project data from the CDW research database.

# **ANNOTATION GUIDELINES**

A dataset of 103 hospital discharge summaries, each concerning a different patient, were randomly selected and annotated for the training and the evaluation of our model. First, the entities “drug name”, “drug strength”, “drug dosage”, “drug form”, “laboratory test name”, “complete laboratory test”, were manually identified in the entire dataset (103 discharge summaries). Following the same annotations rules as proposed by the national NLP clinical challenges 2022 [44] the qualifiers “negation” (drug not taken), “temporality” (present, past, future), “action” (interrupting, tapering the dosage etc.) and “certainty” (“the patient would take...”) were then assigned to each drug name in the entire dataset (103‌‌‌ discharge summaries). Therefore, all 103 discharge summaries were annotated for NER and qualification, with 83 randomly selected for training and the remaining 20 used for testing. These 20 test discharge summaries were further annotated for measurement extraction: values, units associated with the laboratory tests were annotated (e.g., “DFG 90.0mL/min”, the value is 90.0, the unit is mL/min). Finally, of these 20 documents, 11 discharge summaries were randomly selected, and standard codes were annotated for each drug name and laboratory test name in the text. The drug names were assigned to a code from the ATC classification system and the laboratory test names were assigned Concept Unique Identifiers (CUIs) of the Unified Medical Language System (UMLS) restricted to the Laboratory Procedure semantic type and SNOMED CT vocabulary [36]. A detailed definition of each label and qualifiers are listed below:

## **Laboratory test data**

Labels:

1. "**Laboratory test name**": it corresponds to the mention of the laboratory test name alone, including with interpretation. ex: "Hb", "HbA1c", "glycated hemoglobin" etc... "anemia", "thrombocytopenia", "hypokalemia".
2. "**Complete laboratory test**": it corresponds to the complete laboratory test statement: laboratory test name + numerical value +/- unit and normal interval if present, or qualitative information ("normal" or "positive/negative) ex: "Hb = 12g/dl", "Positive antinuclear factor", "2% blasts", "Normal liver panel", "Positive micraoalbuminuria".

Qualifiers:

None

NB:

- NO annotation in "Laboratory test name" of Grouping of results not commonly defined: "Autoimmune workup", "Infectious work-up” “Urinary dipstick”
- NO annotation in "Complete laboratory test" of biological anomalies constituting a pathology: "Chronic renal failure stage 3B", "HyperPTH","Cholestasis.”
- NO annotation in "Complete laboratory test" of recommendation thresholds e.g., "LDL target < 0.7g/L", "transfusion if platelets < 10,000/mm3" etc., but annotation of bio for the latter.
- NO annotation in "Complete laboratory test" of information to be interpreted such as "BNP increased", "leukocytes strong" or "ASAT at 3N" where no clear cut-off is implied (so basically, we just annotate "normal/normal" or "positive/negative".
- Annotate "Laboratory test name" as completely as possible: "Blood gas in ambient air", "Hyposidermic microcytic anemia", "Average blood glucose" / "Maximum creat" / "Peak troponin".
- For haemostasis, annotate "Laboratory test name": "Patient Quick Time" and "Control Quick Time" and the ratio between the 2.
- For "Laboratory test name" whose title is long and cut off in the CR (e.g., "Polynuclear Eo 1") and whose full form is indicated lower down in the CR (e.g., "1 Polynuclear Eosinophil"), annotate "Laboratory test name" the 2 parts.

## **Drug data**

Labels:

1. "**Drug name**": Annotation of drugs including the therapeutic classes "insulin", "corticosteroid therapy", "chemotherapy" but not, for example, generic terms such as "dual therapy" or vaccination.
2. "**Drug form**": what is the galenic form? pill, capsule, tablet, syrup, patch, infusion etc...
3. "**Drug strength**": unit dosage of the drug: 500mg, 1g, etc...
4. "**Drug dosage**": "1g/d", "1/d", "0.5mg/kg/day": complete dosage.

NB1: by default, if not clarified (e.g. Amoxicillin 3g/d), use the annotation "dosage" and not "strenght" (which is more related to the galenic)

Qualifiers:

1. **Action**:

- "Start": medication started, prescriptions also taken into account, e.g. "I'm prescribing corticosteroids" etc... "relay by...", "switch for...", "restart of ...".

- "Stop": medication stopped or suspended.

- "Unique_dose": single dose, e.g., "cure of ...", "vaccination with...".

- "Increase": dose increase

- "Decrease": decrease in drug dose.

- "OtherChange": other change

NB1: if it's just a list of treatment entries and exits, there's no mention of action.

NB2: for temporary treatments (e.g., "Amoxicilin from July 3 to 10"), write "Start" and "Stop" at the same time; the same applies to one-off doses such as "uvedose 1 ampoule now and 1 in 3 months".

1. **Certainty**:

- "Certain": the drug is given/has been taken with certainty. By default, the input/output treatment list is certain, in the same way as for a prescribed drug.

- "Conditional": the drug is prescribed "if needed" (if fever, if diarrhea...).

- "Hypothetical": the treatment or its intake is hypothetical: "the patient would have been treated with Doliprane"...

1. **Temporality**:

- "Past": treatment taken in the past, including written in the present tense in the history of the illness, with dates in the past (e.g., 05/10/2002: patient on corticosteroids) unless the treatment is clearly mentioned as "long-term".

- "Present": treatment currently being taken, including list of treatments taken and withdrawn.

- "Future": treatment prescribed for a future date.

1. **Negation**:

- Yes/no: medication taken or not, given or not. "Contraindication to NSAIDs", "no need for contraception".

# **TERMINOLOGIES DICTIONARIES**

| **Label** | **No. unique standard codes** | **No. corresponding terms** |
| --- | --- | --- |
| **Laboratory test name** | 10 370 CUI | 52 240 |
| **Drug name** | 6 707 ATC codes | 75 631 |

**Table S2. Description of the knowledge dictionaries for drug names and laboratory tests.** The knowledge dictionary for drugs aggregates two open-source resources: the UMLS [20], restricted to the French ATC [37] vocabulary, and the Unique Drug Interoperability Repository created by the French National Agency for Medicines and Health Products Safety [52]. For laboratory tests, the knowledge dictionary includes all French and English synonyms from the UMLS [20], restricted to the Laboratory Procedure semantic type, and terms from the SNOMED CT US Edition vocabulary [36].

# **NER - RULE-BASED PERFORMANCE**

| **Label** | **No. of entities** | **Precision** | **Recall** | **F1-score** |
| --- | --- | --- | --- | --- |
| **Drug name** | 585 [726-459] | 73.8 [65.9-80.4] | 65.3 [55.5-73.9] | 69.3 [61.1-76.0] |
| **Laboratory test name** | 1292 [1598-1009] | 58.5 [53.7-63.1] | 33.4 [28.6-38.0] | 42.6 [38.0-46.4] |
| **Overall** | 1877 [2249-1555] | 64.8 [61.1-68.7] | 43.4 [39.8-46.9] | 52.0 [48.9-54.8] |

**Table S3. Performance of the rule-based method for named entity recognition on AP-HP discharge summaries.** The method was tested on 20 discharge summaries. Each result was bootstrapped by discharge summary to provide a 95% confidence interval given inside the brackets.

# **NER - COMPARE PERFORMANCE PERFORMANCE WITH DIFFERENT PRETRAINED BERT**

| **Fine-tuned model** | | **DrBert [51]** | | | **CamemBert-EDS [48]** | | |
| --- | --- | --- | --- | --- | --- | --- | --- |
| **Label** | **No. of entities** | **Precision** | **Recall** | **F1-score** | **Precision** | **Recall** | **F1-score** |
| **Laboratory test name** | 1292  [1592-999] | 86.4  [82.4-89.8] | 86.3  [83.6-88.7] | 86.3  [83.3-89.0] | **89.5**  **[86.9-91.9]** | **91.0**  **[88.5-93.1]** | **90.3**  **[88.0-92.2]** |
| **Complete laboratory test** | 1041  [1311-786] | 81.4  [77.3-84.7] | 76.5  [71.8-80.0] | 78.9  [74.8-82.0] | **83.2**  **[79.4-86.8]** | **82.0**  **[78.1-85.5]** | **82.6**  **[79.0-86.0]** |
| **Drug name** | 585  [728-454] | 86.1  [80.7-91.3] | 85.0  [80.1-89.1] | 85.5  [80.9-89.2] | **90.7**  **[85.4-95.4]** | **92.0**  **[87.7-95.4]** | **91.3**  **[86.9-94.8]** |
| **Drug dosage** | 276  [369-192] | 79.5  [73.6-85.2] | 77.2  [70.9-82.5] | 78.3  [72.4-83.5] | **87.1**  **[84.1-90.8]** | **87.7**  **[84.0-92.1]** | **87.4**  **[84.1-91.3]** |
| **Drug form** | 170  [247-107] | 82.6  [70.7-93.8] | 83.5  [75.0-89.9] | 83.0  [74.0-91.0] | **86.3**  **[77.5-93.7]** | **92.9**  **[87.4-97.7]** | **89.5**  **[82.7-95.1]** |
| **Drug strength** | 130  [194-76] | 83.7  [72.9-90.4] | 86.9  [80.6-91.0] | 85.3  [77.0-90.1] | **93.9**  **[86.5-98.7]** | **95.4**  **[91.4-98.7]** | **94.7**  **[89.8-98.1]** |
| **Overall** | 3494  [4180-2879] | 84.1  [81.6-86.5] | 82.3  [79.8-84.5] | 83.2  [80.9-85.3] | **87.7**  **[85.5-89.8]** | **88.5**  **[86.5-90.2]** | **88.1**  **[86.1-89.9]** |

**Table S4. Performance of the NER algorithms built upon DrBert [51] or CamemBert-EDS [48] evaluated on AP-HP discharge summaries.** The methods were tested on 20 discharge summaries. Each result was bootstrapped by discharge summary to provide a 95% confidence interval given inside the brackets. Best scores in bold.

| **Fine-tuned model** | | **CamemBert-Base [49]** | | | **CamemBert-Bio [50]** | | |
| --- | --- | --- | --- | --- | --- | --- | --- |
| **Label** | **No. of entities** | **Precision** | **Recall** | **F1-score** | **Precision** | **Recall** | **F1-score** |
| **Laboratory test name** | 1292  [1592-999] | 86.2  [82.8-89.0] | 86.2  [82.6-89.0] | 86.2  [83.0-88.8] | **89.5**  **[87.5-91.4]** | **88.4**  **[85.7-90.5]** | **88.9**  **[86.7-90.8]** |
| **Complete laboratory test** | 1041  [1311-786] | 79.1  [72.0-85.3] | 78.7  [73.6-83.6] | 78.9  [73.0-84.3] | **83.6**  **[79.6-87.6]** | **79.5**  **[75.6-83.0]** | **81.5**  **[78.0-85.0]** |
| **Drug name** | 585  [728-454] | 85.7  [80.2-90.8] | 88.4  [83.1-92.7] | 87.0  [81.9-91.2] | **90.0**  **[84.8-94.7]** | **89.6**  **[83.9-94.3]** | **89.8**  **[84.8-93.9]** |
| **Drug dosage** | 276  [369-192] | **88.9**  **[85.4-93.3]** | 87.0  [83.5-91.3] | **87.9**  **[84.9-91.6]** | 86.8  [83.9-90.4] | 85.9  [82.1-90.2] | 86.3  [83.3-90.1] |
| **Drug form** | 170  [247-107] | 82.0  [69.7-92.7] | 85.9  [77.2-93.3] | 83.9  [73.5-92.4] | **83.2**  **[72.4-92.2]** | **90.0**  **[82.9-95.6]** | **86.4**  **[77.7-93.4]** |
| **Drug strength** | 130  [194-76] | **91.7**  **[82.3-98.0]** | 93.8  [88.2-98.3] | 92.8  [86.0-97.4] | 90.5  [80.7-97.1] | **95.4**  **[91.4-98.5]** | **92.9**  **[86.7-96.8]** |
| **Overall** | 3494  [4180-2879] | 84.2  [81.4-87.0] | 84.7  [82.1-87.0] | 84.4  [81.9-86.9] | **87.4**  **[85.3-89.2]** | **86.1**  **[83.8-88.0]** | **86.7**  **[84.6-88.4]** |

**Table S5. Performance of the NER algorithms built upon CamemBert-Base [49] or CamemBert-Bio [50] evaluated on AP-HP discharge summaries.** The methods were tested on 20 discharge summaries. Each result was bootstrapped by discharge summary to provide a 95% confidence interval given inside the brackets. Best scores in bold.

# **NER – MODEL ARCHITECTURE**

| **Architecture parameters** | | |
| --- | --- | --- |
|  | Pretrained model | CamemBert-EDS [48] |
|  | Transformer window | 128 tokens |
|  | Transformer strid | 96 tokens |
|  | CNN^a^ kernel size | 3 |
|  | CRF^b^ window | 40 tokens |
| **Fine-tuning hyperparameters** | | |
|  | Batch size | 2000 words |
|  | Learning rate | 5.10^–5^ |
|  | Optimizer | AdamW |
|  | Warmup steps | 400 |

^a^CNN: convolution neural network.

^b^CRF: conditional random field.

**Table S6.** Model architecture and fine-tuning hyperparameters for the named entity recognition system. Key parameters of the named entity recognition model’s architecture, including transformer configuration, convolution neural network kernel size, and conditional random field window. It also details the hyperparameters used during fine-tuning, such as batch size, learning rate, optimizer, and warmup steps.

# **NORMALIZE - COMPARE PERFORMANCE WITH DIFFERENT METHODS**

| **Label** | **Model** | **No. of entities** | **Precision** | **Recall** | **F1-score** |
| --- | --- | --- | --- | --- | --- |
| **Laboratory test name** | **Rule-based**  **(JW distance) [53]** | 356 [200, 537] | 71.5  [62.2, 76.7] | 55.6  [46.0, 62.5] | 62.6  [53.0, 68.8] |
|  | **Rule-based**  **(LEV distance) [54]** | 356 [202, 562] | 61.6  [51.8, 67.6] | 58.1  [49.3, 66.3] | 59.8  [50.4, 66.8] |
|  | **CODER-all [33]** | 356 [208, 549] | **92.6**  **[89.8, 95.6]** | **73.9**  **[68.4, 79.7]** | **82.2**  **[77.9, 86.4]** |
|  | **SapBERT-all [32]** | 356 [193, 539] | 73.3  [68.7, 77.0] | 73.3  [68.7, 77.0] | 73.3  [68.7, 77.0] |
| **Drug name** | **Rule-based**  **(JW distance) [53]** | 312 [213, 424] | 95.9  [93.8, 97.6] | **90.1**  **[86.9, 92.5]** | **92.9**  **[90.3, 94.8]** |
|  | **Rule-based**  **(LEV distance) [54]** | 312 [202, 424] | 92.1  [89.4, 94.3] | 89.7  [87.2, 91.9] | 90.9  [88.3, 93.0] |
|  | **CODER-all [33]** | 312 [212, 433] | **96.7**  **[94.1, 98.9]** | 83.7  [79.8, 87.3] | 89.7  [86.8, 92.3] |
|  | **SapBERT-all [32]** | 312 [213, 426] | 84.0  [79.7, 88.2] | 84.0  [79.7, 88.2] | 84.0  [79.7, 88.2] |

# **Table S7. Performance of the normalization algorithms with rule-based method or BERT-based method evaluated on AP-HP discharge summaries.** The methods were tested on 20 discharge summaries. Each result was bootstrapped by discharge summary to provide a 95% confidence interval given inside the brackets.

# **STUDY COHORT - DISTRIBUTION OF AGES AND ADMISSION START DATES**

**
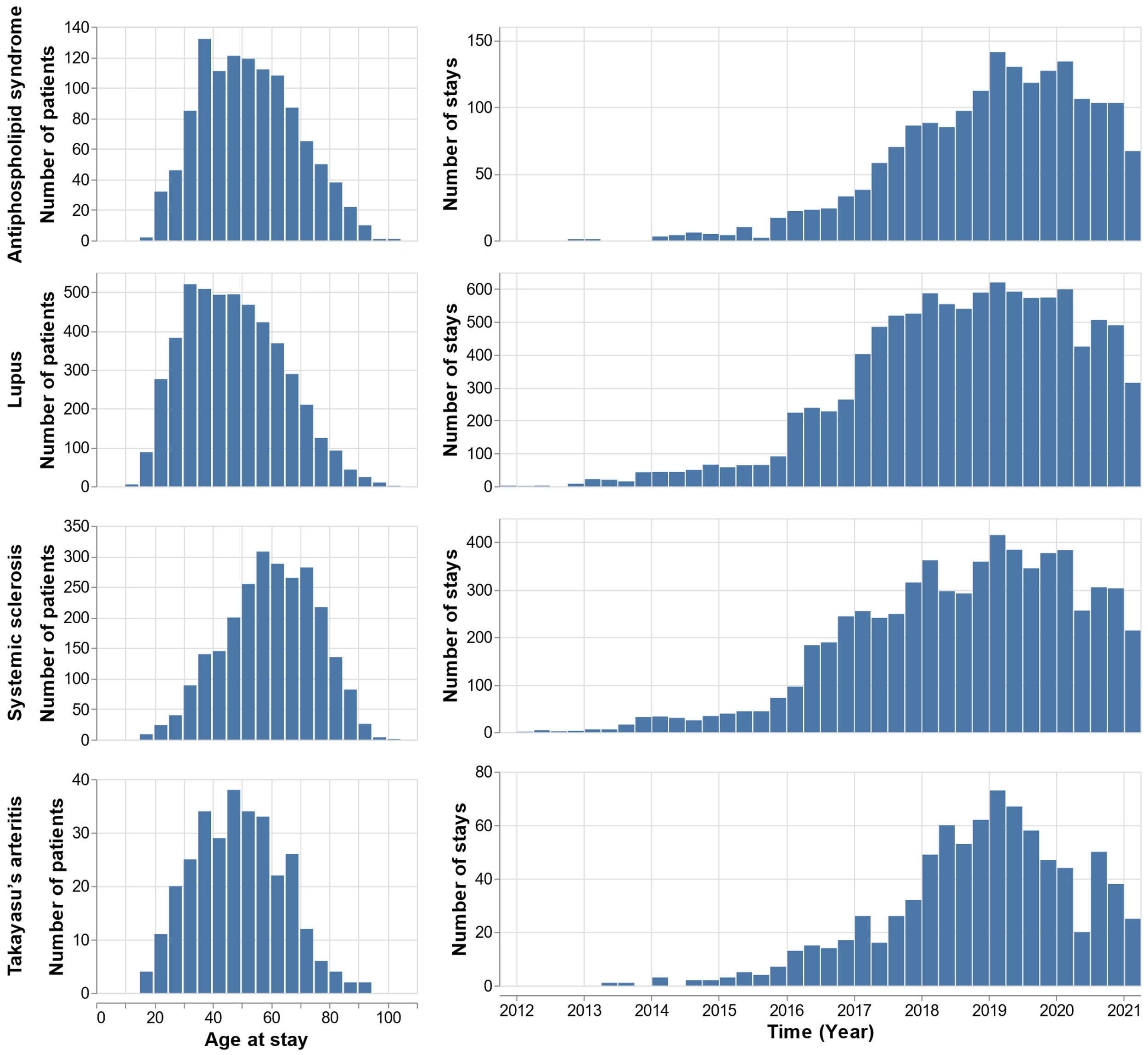
**

**Figure S1. Age distributions and hospital stays over time.** The left column displays histograms of the age distribution of patients in five-year intervals. The right column shows the number of hospitalizations over time from 2012 to 2021 in quarterly intervals.

# **NER - PERFORMANCE ON THE QUAERO DATASET [55]**

| **Label** | **No. of entities** | **Precision** | **Recall** | **F1-score** |
| --- | --- | --- | --- | --- |
| *ANAT* | 510 [562-459] | 79.2 [75.5-83.0] | 69.6 [65.7-73.8] | 74.1 [70.9-77.4] |
| *CHEM* | 342 [392-297] | 78.3 [73.0-83.2] | 68.4 [63.8-73.0] | 73.0 [68.9-76.9] |
| *DEVI* | 35 [50-21] | 44.4 [20.0-69.2] | 22.9 [9.1-39.4] | 30.2 [12.8-47.8] |
| *DISO* | 995 [1069-922] | 74.1 [70.6-77.5] | 52.1 [49.3-55.0] | 61.2 [58.3-63.9] |
| *GEOG* | 51 [66-37] | 80.4 [67.4-92.7] | 72.6 [60.0-84.4] | 76.3 [65.1-86.6] |
| *LIVB* | 321 [364-280] | 82.3 [77.8-86.4] | 72.3 [67.6-77.2] | 77.0 [73.0-80.8] |
| *OBJC* | 39 [54-25] | 75.0 [50.0-100.0] | 23.1 [10.4-38.1] | 35.3 [17.4-52.4] |
| *PHEN* | 49 [64-35] | 76.2 [54.5-94.1] | 32.7 [18.2-47.7] | 45.7 [28.0-61.3] |
| *PHYS* | 159 [192-129] | 56.8 [46.3-67.0] | 34.0 [27.1-41.2] | 42.5 [34.8-49.6] |
| *PROC* | 610 [667-554] | 70.7 [66.7-74.3] | 62.0 [58.1-65.7] | 66.0 [62.5-69.3] |
| *Overall* | 3111 [3241-2985] | 75.0 [73.2-76.7] | 59.2 [57.6-60.8] | 66.2 [64.7-67.6] |

**Table S8. Performance of the model for named entity recognition on MEDLINE corpus.** The model was trained on 1664 annotated titles and tested on 833 titles. Each result was bootstrapped by title to provide a 95% confidence interval given inside the brackets.

| **Label** | **No. of entities** | **Precision** | **Recall** | **F1-score** |
| --- | --- | --- | --- | --- |
| *ANAT* | 182 [234-136] | 90.5 [84.3-95.8] | 83.5 [79.1-88.0] | 86.9 [82.9-90.6] |
| *CHEM* | 885 [1034-722] | 80.1 [73.0-86.4] | 69.4 [61.7-78.0] | 74.3 [67.6-81.3] |
| *DEVI* | 100 [154-50] | 91.7 [84.2-100.0] | 33.0 [13.3-51.4] | 48.5 [23.4-65.9] |
| *DISO* | 341 [454-237] | 65.8 [55.8-77.6] | 50.2 [41.2-60.9] | 56.9 [47.5-67.9] |
| *GEOG* | 14 [24-6] | 92.3 [73.3-100.0] | 85.7 [63.6-100.0] | 88.9 [70.6-100.0] |
| *LIVB* | 266 [309-225] | 87.1 [82.8-91.5] | 81.2 [72.8-88.1] | 84.1 [78.1-88.8] |
| *OBJC* | 53 [88-23] | 41.2 [12.5-81.8] | 13.2 [4.2-30.4] | 20.0 [6.5-42.1] |
| *PHEN* | 33 [62-12] | 52.9 [26.7-78.6] | 27.3 [10.5-69.2] | 36.0 [16.0-61.5] |
| *PHYS* | 85 [119-54] | 76.7 [66.3-88.4] | 65.9 [47.7-78.1] | 70.9 [59.5-77.4] |
| *PROC* | 269 [322-219] | 78.1 [69.9-86.2] | 70.3 [62.1-79.2] | 74.0 [68.3-79.6] |
| *Overall* | 2228 [2525-1944] | 79.3 [75.8-82.6] | 65.5 [59.7-71.7] | 71.7 [67.5-76.1] |

# **Table S9. Performance of the model for named entity recognition on EMEA corpus.** The model was trained on 6 annotated documents and tested on 3 documents. Each result was bootstrapped by document to provide a 95% confidence interval given inside the brackets.

# **QUALIFICATION - PERFORMANCE ON THE APHP DATASET**

| Qualifiers | Number of entities (95% CI) | Precision (95% CI) | Recall (95% CI) | *F*_1_-score (95% CI) |
| --- | --- | --- | --- | --- |
| *Action* | 179 (267-111) | 78.3 (66.4-87.1) | 76.5 (69.0-82.5) | 77.4 (68.5-83.4) |
| *Certainty* | 53 (85-26) | 82.1 (56.8-100.0) | 60.4 (39.5-82.1) | 69.6 (50.0-86.2) |
| *Negation* | 42 (56-27) | 87.5 (76.3-95.7) | 83.3 (69.4-97.2) | 85.4 (75.0-94.3) |
| *Temporality* | 216 (303-144) | 77.7 (68.7-84.8) | 83.8 (74.0-91.5) | 80.6 (72.7-86.3) |
| *Overall* | 490 (661-348) | 79.1 (73.3-84.0) | 78.6 (71.8-84.3) | 78.8 (73.2-83.6) |

**Table S10.** Performance of the model for qualification on University Hospitals of Greater Paris (Assistance Publique-Hôpitaux de Paris) discharge summaries. The model was trained on 83 annotated discharge summaries and tested on 20 discharge summaries. Each result was bootstrapped by discharge summary to provide a 95% CI given inside the brackets. A value for each qualifier was assigned to each “drug name” entity. However, these values were not uniformly distributed. For each qualifier, we defined a “default value,” for example, for the certainty qualifier, most entities were “certain.” To evaluate the model’s ability to detect unusual values (for example, a “conditional” certainty), default values were considered as negative classifications. Thus, for each qualifier, a true positive result was achieved when a predicted value is not a default value and matched the qualifier’s value of the gold entity.

# **MEASUREMENT EXTRACTION - PERFORMANCE ON THE APHP DATASET**

| Number of entities (95% CI) | Precision (95% CI) | Recall (95% CI) | *F*_1_-score (95% CI) |
| --- | --- | --- | --- |
| 789 (557-1038) | 98.2 (96.9-99.3) | 95.2 (91.2-97.8) | 96.7 (94.5-98.1) |

**Table S11.** Performance of the model for measurement extraction. This rule-based algorithm was evaluated on 20 annotated discharge summaries. Each result was bootstrapped by discharge summary to provide a 95% CI given inside the brackets.

# **NORMALIZATION - PERFORMANCE ON THE APHP DATASET AND QUAERO DATASET [55]**

| Label | Dataset | Number of entities (95% CI) | Precision (95% CI) | Recall (95% CI) | *F*_1_-score (95% CI) |
| --- | --- | --- | --- | --- | --- |
| *Laboratory test name* | | | | | |
|  | *Discharge summaries* | 356 (199-551) | 92.6 (89.7-95.6) | 73.9 (68.6-79.6) | 82.2 (77.8-86.4) |
|  | *EMEA* | 20 (12-30) | 89.5 (60.0-100.0) | 85.0 (53.3-100.0) | 87.2 (57.9-100.0) |
|  | *MEDLINE* | 81 (74-88) | 88.9 (80.3-96.4) | 59.3 (48.1-70.6) | 71.1 (60.6-80.6) |
| *Drug name* | | | | | |
|  | *Discharge summaries* | 312 (204-425) | 95.9 (93.9-97.7) | 90.1 (86.6-92.5) | 92.9 (90.2-94.8) |
|  | *EMEA* | 734 (576-918) | 92.2 (89.8-94.6) | 85.3 (79.9-89.8) | 88.6 (85.2-91.6) |
|  | *MEDLINE* | 334 (318-350) | 88.3 (84.6-91.9) | 81.4 (77.0-85.6) | 84.7 (80.7-88.5) |

**Table S12.** Performance of the models for normalization task on University Hospitals of Greater Paris (Assistance Publique-Hôpitaux de Paris) discharge summaries and Quaero FrenchMed corpus. The models were evaluated on 11 annotated discharge summaries from University Hospitals of Greater Paris (Assistance Publique-Hôpitaux de Paris), 3 documents from EMEA, and 833 titles from MEDLINE. Each result was bootstrapped by document to provide a 95% CI given inside the brackets.

# **STANDARD CODES FOR STUDIED TESTS AND DRUGS**

| **Laboratory test** | **CUIs (SNOMED CT US Edition [36])** |
| --- | --- |
| **Anti-cardiolipin antibody** | C0201535, C0455311, C0523546, C0455310 |
| **Anti-B2GP1 antibody** | C1295005, C1303280, C1446135, C1446133 |
| **Lupus anticoagulant** | C0455328, C1142517, C1277823, C0522828, C1096078 |
| **Antinuclear antibody** | C0587178, C1271804, C1273464, C1277811, C0016318 |
| **Anti-DNA antibodies** | C1262035, C0282056, C0201350, C2940788 |
| **Anti-Sm** | C0201357 |
| **Anti-RNA Pol III antibody** | C1295034, C1294732, C1294733 |
| **Anti-SCL 70** | C0523317 |
| **Anti-centromere antibody** | C0201361, C1276016 |
| **Hemoglobin** | C0518015 |
| **CRP** | C0201657 |

**Table S13.** List of Concept Unique Identifiers (CUIs) of the Unified Medical Language System (UMLS) restricted to the Laboratory Procedure semantic type and SNOMED CT vocabulary for the studied autoantibodies and laboratory tests.

| **Drug** | **ATC codes [37]** |
| --- | --- |
| **VKA** | B01AA, B01AA01, B01AA02, B01AA03, B01AA04, B01AA07, B01AA08, B01AA09, B01AA10, B01AA11, B01AA12 |
| **Heparin** | B01AB, B01AB01, B01AB02, B01AB04, B01AB05, B01AB06, B01AB07, B01AB08, B01AB09, B01AB10, B01AB11, B01AB12, B01AB51 |
| **Oral anticoagulant** | B01AF02, B01AF01 |
| **Systemic glucocorticoids** | H02AB, H02AB01, H02AB02, H02AB03, H02AB04, H02AB05, H02AB06, H02AB07 H02AB08, H02AB09, H02AB10, H02AB11, H02AB12, H02AB13, H02AB14, H02AB15, H02AB17, H02AB18 |
| **Cyclophosphamide** | L01AA01 |
| **Mycophenolate Mofetil** | L04AA06 |
| **Rituximab** | L01XC02, L01FA01 |
| **Belimumab** | L04AA26 |
| **Methotrexate** | L04AX03, L01BA01 |
| **Hydroxychloroquine** | P01BA02, M01CA |
| **Prevenar 13 vaccine** | J07AL02 |
| **Pneumovax vaccine** | J07AL01 |
| **Influenza vaccine** | J07BB, J07BB01, J07BB02, J07BB03, J07BB04 |

**Table S14.** List of codes from the Anatomical Therapeutic Chemical (ATC) classification system for the studied drug treatments.
